# Development of an adaptive test of musical scene analysis abilities for normal-hearing and hearing-impaired listeners

**DOI:** 10.3758/s13428-023-02279-y

**Published:** 2023-11-13

**Authors:** Robin Hake, Michel Bürgel, Ninh K. Nguyen, Alinka Greasley, Daniel Müllensiefen, Kai Siedenburg

**Affiliations:** 1 Department of Medical Physics and Acoustics, University of Oldenburg, Oldenburg, Germany; 2 School of Music, University of Leeds, Leeds, UK; 3 Department of Psychology, Goldsmiths, University of London, London, UK; 4 Hanover Music Lab, Hochschule Für Musik, Theater und Medien, Hannover, Germany

**Keywords:** Auditory scene analysis, Musical abilities, Hearing impairment, Music perception

## Abstract

Auditory scene analysis (ASA) is the process through which the auditory system makes sense of complex acoustic environments by organising sound mixtures into meaningful events and streams. Although music psychology has acknowledged the fundamental role of ASA in shaping music perception, no efficient test to quantify listeners’ ASA abilities in realistic musical scenarios has yet been published. This study presents a new tool for testing ASA abilities in the context of music, suitable for both normal-hearing (NH) and hearing-impaired (HI) individuals: the adaptive Musical Scene Analysis (MSA) test. The test uses a simple ‘yes–no’ task paradigm to determine whether the sound from a single target instrument is heard in a mixture of popular music. During the online calibration phase, 525 NH and 131 HI listeners were recruited. The level ratio between the target instrument and the mixture, choice of target instrument, and number of instruments in the mixture were found to be important factors affecting item difficulty, whereas the influence of the stereo width (induced by inter-aural level differences) only had a minor effect. Based on a Bayesian logistic mixed-effects model, an adaptive version of the MSA test was developed. In a subsequent validation experiment with 74 listeners (20 HI), MSA scores showed acceptable test–retest reliability and moderate correlations with other music-related tests, pure-tone-average audiograms, age, musical sophistication, and working memory capacities. The MSA test is a user-friendly and efficient open-source tool for evaluating musical ASA abilities and is suitable for profiling the effects of hearing impairment on music perception.

**Supplementary Information:**

The online version contains supplementary material available at 10.3758/s13428-023-02279-y.

## Introduction

A necessary foundation for developing an advanced understanding of music perception is auditory scene analysis (ASA)—the process by which the auditory system organises the acoustic environment into separate coherent events and streams (Bregman, 1990). This process is essential for allowing listeners to make sense of complex sounds and to distinguish between different sources or elements within an auditory scene. ASA is critical for normal hearing in natural environments, but it is also critical for music perception, because disentangling simultaneous streams of sound (e.g., an oboe within an orchestra, or a tenor voice within a choir) is a key part of music appreciation and can be difficult, particularly for hearing-impaired individuals (Greasley et al., 2020; Madsen & Moore, 2014; Siedenburg et al., 2021). Even though music psychology has long acknowledged the fundamental role of ASA in shaping music perception, at the same time, no efficient and ecologically valid test to precisely quantify listeners’ ASA ability in realistic musical scenarios has yet been published.

Prior research on ASA has largely employed atomistic approaches, which involve methods to examine auditory perception by isolating and analysing individual components or features within auditory scenes, simplifying complex stimuli to understand the underlying perceptual mechanisms (e.g., Bregman & Campbell, 1971; Micheyl et al., 2013). For example, one study by Bey and McAdams (2002) explored the role of schema-based processes in streaming using a melody recognition task with two unfamiliar six-tone sequences. Their findings revealed that performance improved with increasing frequency difference between target and distractor tones, and listeners performed better when the target alone was played first, but only when there was a difference in mean frequency between the target and distractor tones. Although approaches such as these have contributed significantly to our understanding of ASA, they may not fully capture the intricacies of real-world listening experiences, particularly in the context of music perception.

In a more recent study, Kirchberger and Russo (2015) developed the adaptive music perception (AMP) test, which includes subtests for metre, harmony, melody, and timbre. Additionally, they introduced the melody-to-chord subtest, which tapped into the realm of ASA by asking participants to identify a target melody that is presented simultaneously with a chordal accompaniment. The task requires participants to segregate the target melody from the background chords, which is an essential aspect of ASA. The AMP incorporates an adaptive testing method that dynamically adjusts the difficulty of test items presented to an individual based on their performance in real time. Nonetheless, the melody-to-chord subtest appeared to be particularly difficult for many participants, with roughly a quarter of normal-hearing (NH) participants and a third of hearing-impaired (HI) participants being unable to complete the task. Moreover, the AMP also uses artificial sound stimuli. Other tests similarly highlighted difficulties with ASA tasks. Siedenburg et al. (2020) adaptively measured signal-to-masker ratio thresholds of NH and HI listeners in a melody and timbre discrimination task, but also needed to discard data from HI listeners who yielded uninterpretable results. Another non-adaptive study, in which participants were asked to track target instruments in a classical piece while also attending to other instruments playing simultaneously, needed to discard data from HI participants as well due to chance performance (Siedenburg et al., 2021). These documented challenges in measuring ASA abilities motivated us to develop an adaptive, computer-driven measurement instrument suitable for assessing ASA abilities in the context of music for individuals with a broad range of listening abilities (that is, suitable for both NH and HI listeners). By employing an ecologically valid methodology that incorporates authentic, recorded music, our aim is to capture the intricate and dynamic nature of auditory scenes, integrating multiple auditory features and cognitive processes for incorporating the overall experience, and providing meaningful measurements for both NH and HI individuals.

HI listeners are known to perform poorly compared to NH listeners on music-related perceptual tasks such as timbre identification (e.g., Emiroglu & Kollmeier, 2008; Siedenburg et al., 2020), rhythm perception, pitch discrimination (Uys & van Dijk, 2011), melodic intonation (Kirchberger & Russo, 2015; Siedenburg et al., 2020), and auditory scene separation (e.g., Bayat et al., 2013). Older HI listeners also experience degraded spatial auditory processing (Akeroyd et al., 2007). While these effects are thought to result from damage to the ear, the auditory nerve, or the nervous system (e.g., Cai et al., 2013), some studies suggest that in fact some of these low performance levels can be explained by the problems associated with ageing and associated cognitive decline (Garami et al., 2020; Goossens et al., 2017; Gordon-Salant & Cole, 2016; Vinay & Moore, 2020). Yet, others argue that neither ageing nor HI can fully explain the observed individual differences, but a combination of these factors (Lentz et al., 2022). Furthermore, a growing body of research deals with the influence of musical sophistication and musical training on auditory perception skills. Even though there is still an ongoing debate as to whether musical training has a beneficial effect on speech perception (e.g., Bidelman & Yoo, 2020; McKay, 2021; Parbery-Clark et al., 2009), several studies have demonstrated a positive link between musical training and music perception and other acoustical abilities (e.g., Madsen et al., 2019; Siedenburg et al., 2020; von Berg et al., 2021; Zendel & Alain, 2012). Moreover, musicians have been reported to outperform non-musicians in basic cognitive tasks, such as those related to working memory (Talamini et al., 2017).

Although there is no clear picture of the causal factors underlying individual differences in ASA, it may be difficult to accurately understand ASA in music without taking into account these differences in the ability to process complex auditory scenes. One method which accounts for the large variability in listeners’ abilities is adaptive testing. In contrast to standard testing procedures, where a fixed set of items is presented to all test-takers regardless of their abilities or performance, in adaptive testing the difficulty level of test items is adjusted based on the responses provided by the test-taker (for an overview see van der Linden & Glas, 2000). This allows the difficulty of the administered items to be tailored to the test-taker's ability level, rather than presenting a fixed set of items that may be too difficult or too easy for some test-takers. There are further benefits of adaptive testing. The comparatively shorter testing times can reduce inattention effects and test bias, making it less likely that test-takers will gain advantage by guessing answers, resulting in more precise measurement estimates compared to standard procedures. This results in greater reliability, even when the testing time is reduced by 50–80% compared to non-adaptive tests (de Ayala, 2009; van der Linden & Glass, 2007; Weiss & Kingsbury, 1984). There are several examples of music-related tests that rely on adaptive testing procedures. Modern tests include measures of beat perception (Harrison & Müllensiefen, 2018), melody discrimination (Harrison et al., 2017), and mistuning perception (Larrouy-Maestri et al., 2019). These tests generally apply item response theory (IRT) models, which is a flexible adaptation approach that can be applied to a wide range of testing situations. However, IRT models require a calibrated item bank with a known difficulty level for all items. We consequently explored factors suitable for manipulating the item difficulty in a task that entails the detection of target sounds in mixtures of popular music.

A common approach for identifying factors that affect the underlying construct of interest is to examine the underlying cognitive processes involved in a task (Embretson, 1983). One key aspect of ASA is the ability to segregate individual sound sources from background signals in a complex auditory mixture. This process involves identifying and grouping together sounds that share similar acoustical characteristics and similarities along perceptual dimensions such as pitch and timbre, and which exhibit similar temporal patterning or common onsets (which are principles of common fate, continuity, similarity, and proximity; see Bregman, 1990). Accordingly, instruments (or vocals) with distinctive timbral qualities are often easier to distinguish in an ensemble because their unique acoustic properties enable them to stand out from the mixture. Bürgel et al. (2021) found that participants’ performance in a detection task was generally dependent on the target’s instrument category, with lead vocals showing a particularly robust attentional salience regardless of low-level acoustic cues. A second relevant component is the acoustical complexity of a mixture. The greater the number of instruments contributing to a musical mixture, the higher the probability of energetic masking (one sound spectrally masks or obscures a quieter sound, making it difficult or impossible to hear) and informational masking (one sound interferes with or disrupts the perception of another sound). Thus, by providing a more complex musical scene, the segregation of individual instruments should become more difficult. Due to the same masking processes, it is also expected that the relative level of the instruments can provide (or hide) important cues for segregating the target sound from the background. Sounds that are louder are typically perceived as more salient than softer sounds. Thus, if the target instrument is presented at a lower level than the background mixture, it may be more difficult to segregate it from the mixture and detect it. Another primitive cue that has been identified as important for encoding perceptual features includes signals’ spatial location, as documented by a large range of studies on spatial release from masking (e.g., Litovsky et al., 2021). The spatial separation between a target sound source and interfering sound sources leads to an improvement in target signal detection. The effect of a priori knowledge about the target location has been studied as well. For instance, in a study by Kidd et al. (2005), participants were asked to identify keywords from a target talker in the presence of two distractors in a setting with spatially separated loudspeakers. Conditions in which a priori knowledge about the target location was provided yielded higher performance than conditions in which no cue was provided.

## The present study

To account for these processes with respect to individual differences among test-takers, we designed a straightforward and simple 'yes–no' task (also known as the 'A–not A' task; see Düvel & Kopiez, 2022) that required participants to decide whether a single target instrument (or lead singing-vocals) was part of a two-second mixture of instruments. In a calibration phase, two online experiments were conducted in order to establish item characteristics that could be used as predictors in an explanatory IRT model. This phase is necessary to fine-tune the test items for the adaptive test version, ensuring they accurately measure the intended construct and provide meaningful results across a broad range of ability levels. Experiment 1 of the calibration phase investigated the influence of the target-to-mixture level ratio (designated as ***LEVEL***), the choice of the target instrument (***TARGET***), and the number of instruments in the mixture (***NUM***) on the test results. Experiment 2 focused on the effect of spatial separation in azimuth (at a stereo width of 0, 90°, 180°) introduced by inter-aural level differences (***ILD***).

### Cognitive model of the Musical Scene Analysis Test (MSA)

In the present task, the full cognitive process model includes the following stages: (1) participants perceive the target instrument as a distinct auditory object; (2) participants store the mental representation of the sound of the target instrument in working memory; (3) participants use bottom-up and top-down processing to separate the target instrument from the background mixture based on its acoustic features and prior knowledge. Within this process, stream segregation is guided according to common principles such as similarity, proximity, continuity, and common fate; (4) participants selectively attend to the target instrument's timbre within the mixture (if present) based on their working memory representation while disregarding other sounds in the mixture. This includes a comparison of all segregated auditory streams to an internal mental representation or template of the target instrument's sound; (5) based on the similarity between the segregated auditory stream and the internal template, the listener decides whether the target instrument is present in the mixture or not. Accordingly, the test imposes demands on various cognitive processes, such as perception, working memory, segregation processes, attention, and decision-making. The task's difficulty is presumably influenced by a combination of factors, including the number and relative prominence of instruments within the mixture, as well as the nature of the target instrument.

Based on the proposed cognitive model of the MSA task and the reviewed literature, we formulated four hypotheses for the calibration phase:

1. Decreasing the target-to-mixture level ratio will make it more difficult for listeners to accurately identify and separate the target sound from the mixture, resulting in lower accuracy.
2. The listener's ability to detect the target instrument within the mixture will decrease as the number of musical instruments in the excerpt increases.
3. There will be differences in detection accuracies for various target instruments. Although the literature provides limited guidance on the direction of these differences, we expect that lead vocals will be the easiest and bass the most difficult to detect, as indicated by Bürgel et al. (2021).
4. An increase in stereo width (induced by inter-aural level differences) will make it easier for listeners to localise and segregate the target sound within the mixture, leading to improved accuracy.

By examining these factors (i.e., ***LEVEL***, ***TARGET***, ***NUM***, ***ILD***), the calibration phase aims to optimise the MSA test items for effectively assessing individual differences in auditory scene analysis abilities in a musical context (irrespective of prior musical experience and hearing impairments). In addition to the online calibration phase, we conducted a validation experiment (experiment 3) in a laboratory context to verify results under controlled conditions. This enabled us to assess the consistency of the MSA test through test–retest reliability analysis and to compare individuals' scene analysis abilities with those in a range of other psychoacoustic and music listening tests.

## Experiment 1: Calibration phase—Part 1

### Methods

#### Test battery

##### Musical Scene Analysis Test (MSA)

**Fig. 1:**
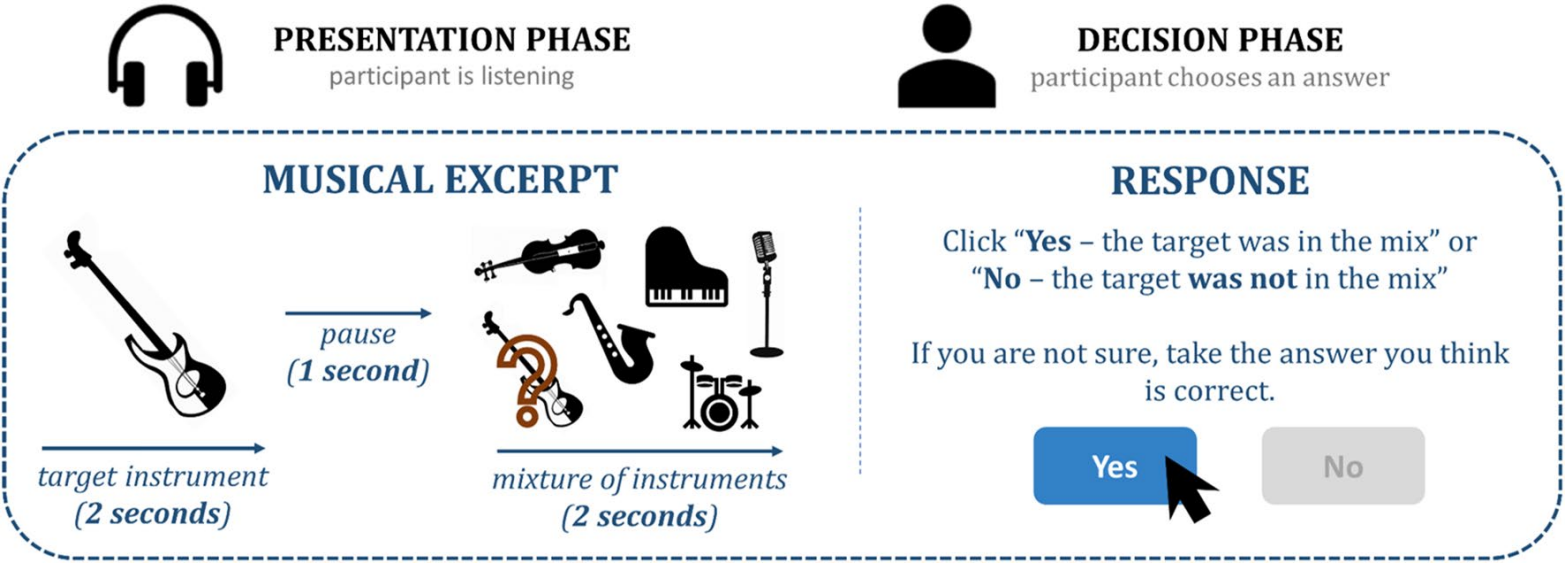
Exemplary schematic illustration of the procedure. Each trial consisted of a two-second excerpt of a single instrument or lead vocals (the target), followed by a one-second gap, and a two-second excerpt with multiple instruments (the mixture). The listener's task was then to decide whether the target instrument was embedded in the mix or not

The MSA is a ‘yes–no’ test that reflects a two-alternative-forced-choice (2-AFC) testing paradigm. The MSA assesses participants’ ASA abilities in realistic musical scenarios by asking participants to detect a single target instrument (or lead vocals) in a mixture of instruments. Each trial consisted of a two-second audio excerpt of a single instrument or voice (the target), followed by a one-second silence, and a two-second excerpt with multiple instruments (the mixture). Participants were then asked to decide whether the target was part of the mixture or not (see Fig. 1 for a schematic illustration).

All excerpts were drawn from an open-source music database (MedleyDB, Bittner et al., 2014, 2016), which consists of real-world multitrack music recordings representing a wide range of musical genres (e.g., pop, rock, world/folk, fusion, jazz, rap, classical). Prior to the extraction, a professional musician with a background in music production meticulously adjusted and post-processed each mix to improve overall audio quality. This process involved refining the balance among individual instruments, fine-tuning volume levels, and minimising signal leakage. The excerpts were generated using the programming environment MATLAB (MathWorks Inc, 2020). In order to identify a set of suitable candidate tracks, the sound levels of each individual instrument within each song were analysed and calculated based on the root-mean-square average over 500 ms time windows for the full duration of the song. If one instrument in the target category and two to six additional instruments had sound levels above − 20 dB relative to the instrument’s maximum sound level in the song, the song qualified as a candidate base track. By setting a minimum sound level threshold of − 20 dB relative to the instrument's maximum sound level in the song, we aim to include only those songs where all chosen instruments are clearly audible.

The candidate list comprised 12,126 potential excerpts, each extracted from a distinct two-second time window within one of the 117 eligible songs in the database. From this list, excerpts were selected pseudo-randomly, with a deliberate effort to minimise duplications of the base song. The selection protocol also ensured an equal distribution of excerpts in terms of the designated target instrument (lead vocals, guitar, bass, or piano) and the number of instruments in the mixture (either three or six). The specific target instruments were selected due to their diverse and widespread accuracy reported in Bürgel et al. (2021), which employed a similar detection task in one of their experimental conditions. The composition of instruments within each mixture was preserved in its original configuration, meaning that it could include a diverse array of instruments such as lead vocals, backing vocals, bass, drums, guitars, keys, piano, percussion, strings, or winds, depending on the base songs used. In half of the mixes, the target instrument did not play in the mixture. In such instances, only excerpts featuring an additional instrument were selected to guarantee the preservation of three or six instrument signals within the musical mixtures for all items. For example, we utilised excerpts originally containing four instruments when a mixture with three instruments was required. Detailed information regarding the specific composition of instruments within each excerpt can be found in the MSA GitHub repository (https://github.com/rhake14/MSA).

Overall, this yielded a 4 (*target instrument categories*) × 2 (*number of instruments in the instrument mixture*) × 2 (*presence of the target in the mixture*) design, for which 160 different excerpts from 98 base songs were compiled. In addition to the experimental factors target instrument (1) and number of instruments in the mixture (2), the first calibration experiment explicitly examined the influences of the target's level ratio in comparison to the mixture (3). To this end, only for those excerpts in which the mix contained the target instrument, four versions were created that varied in their target-to-mixture level ratio (that is, 0, − 5, − 10, − 15 dB). Overall, a total of 400 items were created for the experimental task. Apart from the manipulation of the target instrument, the musical material in the excerpts was left unchanged (i.e., only excerpts were chosen in which the number of instruments corresponded to the desired condition). A logarithmic fade-in and fade-out with a duration of 200 ms was applied to the beginning and end of the audio signals. To allow for use with an online testing platform, all stimuli were converted from WAV format to MP3 with a bit rate of 320 kbit/s stereo (i.e., perceptually lossless compression). All resources, including the MSA test, task description, stimulus details, and example excerpts, are available on the project's GitHub repository (https://github.com/rhake14/MSA).

##### Degree of hearing impairment

Participants were asked to fill out an adaptation of the HAfM (Hearing Aids for Music) National Survey on hearing impairment (e.g., Greasley, 2022). These questions aimed at assessing the type and degree of hearing impairment. Participants were asked ‘Do you feel you have a hearing loss?’ and were able to respond with five options ranging from: ‘No, I do not feel that I have a hearing loss’ to ‘Yes, I have the feeling of being profoundly hearing impaired’. For each option, a short description was given (e.g., ‘Yes, I have the feeling of being mildly hearing impaired: When I am talking to a person in a quiet room, I can usually understand a conversation. In noisy situations (e.g., in a pub) and in group conversations, I sometimes have problems understanding speech.’). See Tables A4 and A5 for the complete self-assessment survey.

##### Goldsmiths Musical Sophistication Index (GMS; Müllensiefen et al., 2014)

The Gold-MSI is a brief, 39-item self-report questionnaire that assesses several aspects of musical expertise. It was designed to capture subscales for active engagement, emotions, musical training, perceptual abilities, and singing abilities. Participants were asked to respond on a a seven-point-Likert scale (1 = completely disagree; 4 = neither agree nor disagree; 7 = completely agree). For both calibration experiments, the two sub-scores for musical training (7 items, for example ‘I engaged in regular, daily practice of a musical instrument (including voice) for ___ years.’) and for perceptual abilities (9 items, for example ‘I can tell when people sing or play out of time with the beat.’) were used. The final composite score ranging from 1 to 7, with 7 being the highest possible score, was generated for each subscale. Both the validated English and German versions that were used and other relevant materials are freely available on the Gold-MSI home page (https://gold-msi.org).

##### Huggins headphone screening (Milne et al., 2020)

This 3-AFC task probes for headphone usage and makes use of a perceptual illusory pitch phenomenon, called the Huggins Pitch. The procedure involves presenting a white noise stimulus to one ear and the same white noise stimulus to the other ear, but 180° phase-shifted over a narrow frequency band at about 600 Hz. A faint tone can then be detected, but only when the stimuli are presented dichotically over headphones. Importantly, when the stimuli are presented to one ear alone or over loudspeakers, the sound is very weak or absent. In order to pass the test, listeners needed to properly identify the tone five out of six times. Participants with severe HI struggled with this task, and since the test was originally calibrated only among NH individuals, only NH participants needed to pass the headphone screening in order not to be excluded from the data analysis. A free demo implementation of the task can be found on GitHub repository (https://github.com/ChaitLabUCL/HeadphoneCheck_Test).

##### Demographics questionnaire

The demographics questionnaire consisted of several items designed to gather participants' background information. Participants were asked to provide information on their age, gender, and educational level. This demographic data helped to characterise the study sample and provided context for interpreting the results of the main experimental measures.

### Procedure

Ethical approval for the study was obtained from the ethics committees at the University of Oldenburg and the University of Leeds. Informed consent was obtained from all participants tested. Two different samples of participants were recruited for experiment 1: For Sample 1, the experiment was conducted using *testable.org*, a web-browser-based application for creating behavioural experiments and surveys online (e.g., Rezlescu et al., 2020). The study was conducted in a single online session, with an average completion time of about 35 min for participants in Sample 1. For those in Sample 2, the average completion time was 10 min. The study was available in both the English and German languages. All participants provided digital consent by signing an electronic form and explicitly agreed, through a checkbox format, to remain in a quiet and distraction-free environment for the duration of the experiment. Participants who usually wore hearing aids were instructed to remove them for the study. All participants were financially compensated based on an hourly rate of €10; participants in Sample 1 received €5, while those in Sample 2 received €2 (or its equivalent in British pounds for UK residents), reflecting the different administration times for each sample group. Prior to the main experiment, a calibration sound was presented, and participants were instructed to adjust the volume of their playback device to a loud but comfortable level. Then the headphone screening was applied. Prior to the main experiment, participants underwent an MSA training session featuring five unique excerpts from the candidate list, which were not included in the main test set. Immediate feedback was provided after each response, and participants had the option of repeating the training phase as often as desired. The training phase was followed by the main experiment, that is, the MSA, where no feedback was given. A total of 160 trials, each presenting a single target-to-mixture level-ratio version of the 160 excerpts, were administered. The order in which the 160 items were presented and the selection of the target-to-mixture level-ratio version was randomised. Participants were allowed to pause at any time, with a recommended pause after half of the trials. After completing the experimental part of the study, a questionnaire regarding personal information including degree of hearing impairment, age, and gender, as well as the two subscales of the Gold-MSI for musical training and perceptual abilities, was administered. The same procedure was followed for Sample 2, but participants were only presented 32 items, with each combination of parameters being given no more than once—effectively reducing the duration of the experiment to approximately 10 min. The rationale for this approach was to obtain a diverse participant sample with individuals exhibiting varied profiles, such as differing hearing abilities, ages, and genders. Accordingly, in Sample 2, we prioritised the diversity of participants over measurement accuracy. For Sample 2, the experiment was conducted using *psychTestR* (Harrison, 2020), an R package for creating web-browser-based behavioural experiments.

#### Participants

**Fig. 2:**
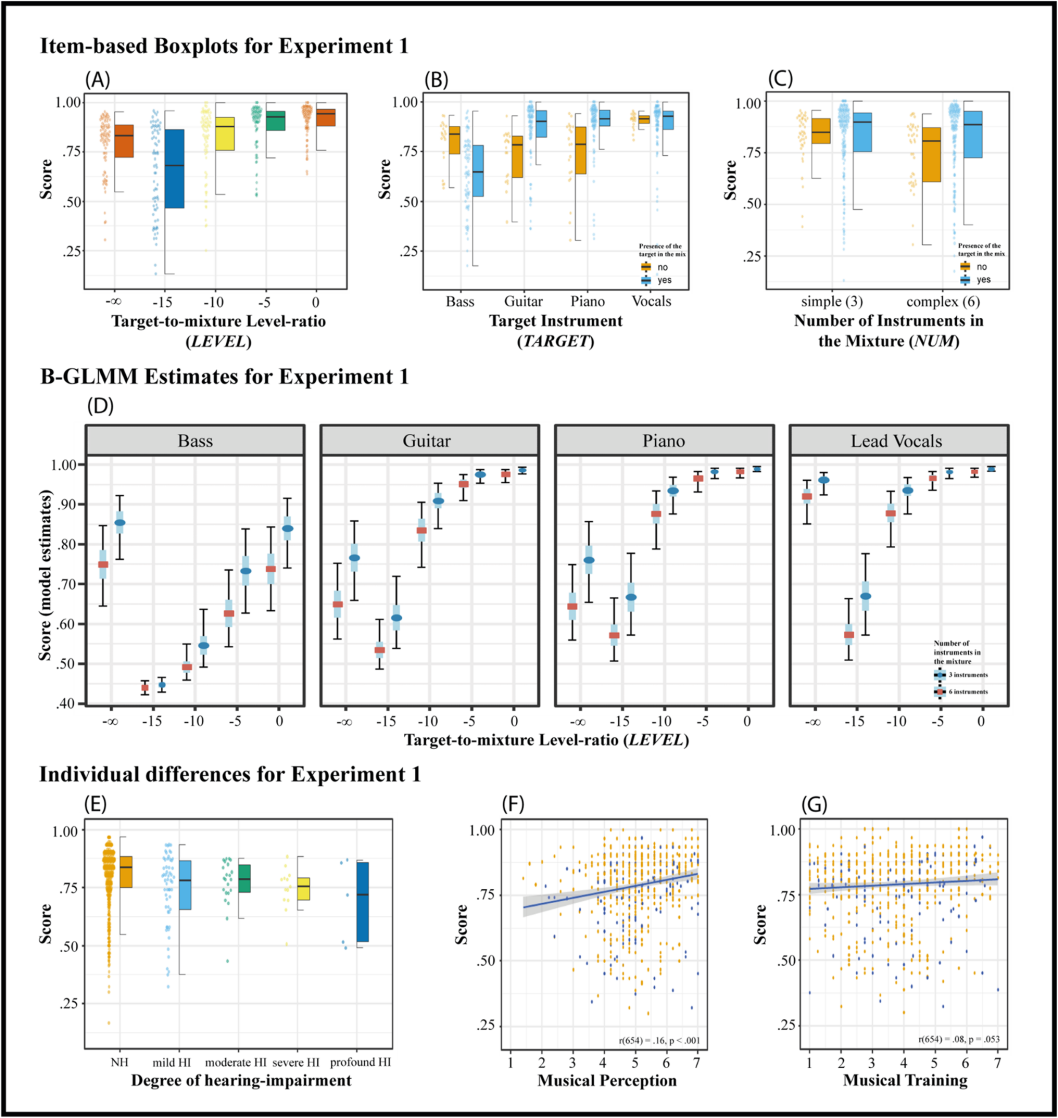
Results of experiment 1. Panels (**A**), (**B**), and (**C**) show the proportion of correct scores of individual test items for each of the parameters employed. Individual dots (left of the boxplots) represent the observed accuracy averaged over participants for each item. Light-blue boxplots correspond to items in which the target was present in the mixture (orange when the target was not present in the mixture). ‘-∞’ corresponds to the items, in which no target was presented in the mixture. Panel (**D**) shows the conditional effects plot of the final B-GLMM (see Table A1). Median estimates with inner 50% (the length of the blue bars around the median) and outer 95% (the length of the black bars around the median) high-density intervals (HDI) of the estimated parameters are shown. HDIs are uncertainty intervals and reflect the probability of the parameter estimate being within the given intervals. Panel (**E**) shows the boxplots of participants’ average accuracy as a function of the degree of hearing impairment. Panels (**F**, **G**) show scatterplots illustrating the relationship between the participants' observed average detection scores and the two subscales of the Gold-MSI (musical training and musical perception). The individual dots represent the score for each participant averaged over all items

The first sample was recruited through a newspaper article, mailing lists, and a call for participation posted at the online job board of the University of Oldenburg, and 126 participants (69 female) took part. Among these, 47 self-reported having at least a mild hearing impairment (14 female; *M* = 61.6 years, *SD* = 16.6), whereas 79 reported having no hearing impairment (55 female; *M* = 26.9 years, *SD* = 9.3). The second sample of participants was recruited via the online market research company SoundOut, located in the United Kingdoms, from which 1078 individuals completed the experiment (*M* = 30.19 years, *SD* = 11.72, 598 female). To ensure some control over the playback conditions during the experiment, participants were instructed to wear two-channel headphones, which was checked with a screening test (Milne et al., 2020). Of the initial sample, 548 individuals failed the headphone screening and were thus excluded. Consequently, the analysis for experiment 1 included a total of 525 NH participants with ages ranging from 18 to 72 years (*M* = 28.6 years, *SD* = 11.02, 274 female) and 131 HI participants with ages ranging from 23 to 82 years (*M* = 40.5 years, *SD* = 20.6, 67 female). The geographical location of participants was as follows: Australia (1), Canada (10), United Kingdom (135), Ireland (1), New Zealand (2), United States (322), Germany (126), and not specified (59). For a detailed overview with respect to the individuals’ degree of hearing impairment (i.e., degree of individuals’ self-rated hearing loss) see Fig. 2E.

### Data analysis

Bayesian generalised logistic mixed-effects models (B-GLMM) were used for the analysis. B-GLMM are powerful and flexible alternatives to more commonly used frequentist approaches. In particular, they are able to account for uncertainty in parameter estimation and can provide stable estimates for categorical variables with many levels and smaller sample sizes with the help of informative prior distributions (e.g., Dienes & Mclatchie, 2018; Stegmueller, 2013). Using (Bayesian) mixed-effect models with a binary dependent variable (correct/incorrect participant response), we can measure how different aspects of a musical excerpt affect its perceptual processing difficulty. We report the median estimates and 95% confidence intervals of the conditional effects for the final Bayesian logistic mixed-effects model. These values were obtained by averaging the conditional effects estimates across the respective factor of interest. By examining credible intervals and comparing the posterior probabilities of different hypotheses, we evaluate the extent to which detection accuracies differ among the respective conditions. The observed descriptive statistics, including the overall results averaged across items, can be found in the accompanying plots, which offer a visual representation of the data and highlight the main patterns observed in the study (see Figs. 2 and 4). Before conducting the mixed-effects analysis, the data were inspected for unexpected response patterns. As a result, a total of 29 items were removed from the final analysis. An individual inspection of these problematic items showed that in some cases, the low target-to-mixture level ratios made the target inaudible. In other cases, backing vocals were so highly similar to the lead vocals that the experimental task was in fact ill-defined. Eight items with lead vocals as the target instrument were also excluded as they showed a success rate of 100% and thus did not provide valuable discriminative information. It should be mentioned that despite some participants scoring below the 50% chance performance level (occurring only in Sample 2), all participant scores were retained in the analysis. The rationale for this decision is twofold: First, the task inherently varies in item difficulty, and excluding low-performing participants could introduce selection bias, potentially underestimating the true challenge posed by certain items and compromising the generalisability of the results. Furthermore, the sample had already been subjected to a rigorous screening process; a significant cohort (*N* = 548) was excluded for failing the headphone screening test, presumably filtering out participants not adequately committed to the task. Retaining all participant scores was thus deemed crucial for preserving the integrity and representativeness of the data.

All analyses were executed in R (v2022.07.2 + 576; RStudio Team, 2020) and the Stan modelling language (v2.21.7; Carpenter et al., 2017), using the package *brms* as an interface from R to *Stan* (v2.18.0; Bürkner, 2017).

#### Bayesian GLMM fitting

We fitted several B-GLMM (Bernoulli family with identity link; estimated using Markov chain Monte Carlo [MCMC] sampling with 35,643 observations, four chains of 6000 iterations, and a warmup of 3000) to predict participants’ performance ***SCORE*** at the level of each individual trial (binary item responses, with 0 = incorrect and 1 = correct). The model was built step by step in a hierarchical way by adding one parameter at a time. This allowed us to evaluate the individual impact of each variable. We first added each parameter to the same model structure separately to gain a first understanding of the general predictive performance of each parameter in isolation (as shown in Table 1).

**Table 1:** Model comparison of all Bayesian (GLM) models of experiment 1

Model	Fixed effects	Random effects	Pareto *k* check	LOOIC	BF
1A	*SCORE* ~ *TARGET*	Random intercept for excerpt and participant	< 0.7	29,191.5	1.84e + 48^*^
1B	*SCORE* ~ *NUM*	Same as A	< 0.7	29,188.9	7.39e + 07^*^
1C	*SCORE* ~ *LEVEL*	Same as A	< 0.7	27,300	Inf^*^
1D	*SCORE* ~ *LEVEL*** + ***TARGET*	Same as A	< 0.7	27,302.4	2.84e − 10^**^
1E	*SCORE* ~ *LEVEL*** + ***TARGET*** + ***NUM*	Same as A	< 0.7	27,295.2	7.12e − 15^**^
1F	*SCORE* ~ *LEVEL*** + ***TARGET:PRESENCE*** + ***NUM:PRESENCE*	Same as A	< 0.7	27,290.45	(Reference for 1D and 1E)^**^
1G	*SCORE* ~ *LEVEL*** + ***TARGET:PRESENCE*** + ***NUM:PRESENCE*	Random slope and random intercept for excerpt and participant	> 0.7^***^	25,590.3	(Excluded)^***^

Model summary (fixed effects) shows how the model was passed to the brms package in R [**SCORE = **binary-coded MSA score; **NUM** = number of instruments in the mix; **LEVEL = **level ratio between target instrument and the mixture; **TARGET = **choice of the target instrument; **PRESENCE** = target instrument is part of the mixture. ‘**:**’ indicates an interaction effect; **LOOIC** = leave-one-out cross-validation information criterion; **BF** = Bayes factor]. Given the structural design of the task, the **LEVEL** parameter always includes an interaction effect with the presence of the target in the mix (**PRESENCE**). ^*^Models 1A, 1B, and 1C were tested against a null model, which includes only random effects but no fixed effects. ^**^Models 1D and 1E were tested against model F, to improve the interpretability of the comparison and to guide model selection. ^***^Pareto *k* values greater than 0.7 indicate potential issues with the model's convergence or sampling efficiency, that is, the model has not captured the underlying structure of the data accurately or there might be issues with the MCMC chains (Vehtari et al., 2019)

We compared three models, each with a single fixed effect, using Bayes factors (BF) in contrast to a null model that included only random effects and no fixed effects. BF is a measure to quantify the evidence for one model over another. A BF of 1 indicates that both the null and alternative models are equally likely, while a BF greater than 1 suggests that the data better support the alternative model. Because the specifications for models 1B, 2B, and 3B differ only in the fixed effect used, we interpret the BF as an indicator of which predictor is most strongly supported by the data for explaining the dependent variable, namely, MSA scores.

Model 1C, which incorporated the ***LEVEL*** as a fixed effect, and model 1A (***TARGET***) provided the strongest evidence compared to the null model (BF_***LEVEL***_ = Inf; BF_***TARGET***_ = 1.84e + 48). This was followed by model 1B, which included the ***NUM*** as a fixed effect (BF = 7.39e + 07). These results suggest that incorporating the ***TARGET*** and especially ***LEVEL*** as fixed effects better explains the observed data than the number of instruments in the mixture (***NUM***), highlighting the potential importance of the choice of the target instrument and the target-to-mixture level ratio for the MSA task. However, it is important to interpret these results with caution, as the Bayes factor only provides relative evidence between models and does not directly quantify the effect size of each fixed effect. Further investigation, including effect size estimation and comparison, is needed to draw more definitive conclusions about the influence of these factors on the outcome variable (as indicated by the medians of the conditional effects and their accompanying density intervals; see Makowski et al., 2019a, 2019b).

According to this analysis, we planned four versions of the model, each one adding a parameter in the order of their hypothesised importance to predict the MSA test scores. We then used the leave-one-out cross-validation information criterion (LOOIC) and BF to provide a statistical measure of the model's predictive performance. ‘LOOIC’ is a model comparison metric derived from the concept of cross-validation. It estimates the predictive accuracy of a model by leaving out one observation at a time, fitting the model to the remaining data, and then predicting the left-out observation. A lower LOOIC value indicates better predictive performance, and the model with the lowest LOOIC value is considered the best (Vehtari et al., 2017). The final model was chosen based on a combination of these measures, to consider the model's relative fit, its complexity, and predictive performance. Model 1G (see Table 1) was excluded from the pool of candidate models, as the Pareto *k* estimate indicates potential issues with the model's convergence or MCMC sampling efficiency. The high BF value suggests that model 1F is likely to best explain the observed data when compared to the other models. Additionally, the low LOOIC value indicates that model 1F offers the optimal balance between its predictive ability and complexity (see Table 1). The final model used (1) ***LEVEL***, (2) ***TARGET***, and (3) ***NUM*** as fixed factors. For each of the factors, an interaction effect with the presence of the target in the mixture (***PRESENCE***) was included. Both excerpt and participant were added as random effects, which allowed the intercept to vary across participants and excerpts. Priors over the guessing and inattention parameters were set as beta distribution (α = 1, β = 1), with an expected lower bound for possible values of 0.4 and an upper bound of 0.6 for the guessing parameter (expected success rate if the participant were to answer randomly), and 0 to 0.1 for the inattention parameter (expected probability of a participant not paying attention), respectively.

### Results

#### Model fit

The final model (1F) convergence and stability of the Bayesian sampling was assessed using Ȓ, which was below 1.01 (Vehtari et al., 2019), whereas the effective sample size (ESS) was above 1000 (Bürkner, 2017). All Pareto *k* estimates were good (*k* < 0.5; Vehtari et al., 2019). For assessing the model's explanatory power we used the classification accuracy of our model, which represents the proportion of correct predictions out of the total number of predictions. Although classification accuracy does not provide a direct measure of explanatory power, it does offer insights into how well the model is performing in predicting binary outcomes. The final model was able to correctly identify 78.3% of the observed response data. When excluding the random-effect information, the three employed fixed-effect factors alone accounted for a reasonable proportion of the model accuracy at 70.7%. The estimates obtained for this model are summarised in Table A1. Overall, the model assumed a guessing parameter of 0.43, which lies within a reasonable scope of the theoretically assumed 0.5. According to the model, the inattention parameter was below 0.01. This suggests inattention effects to be negligible for the short test durations employed in the present research. A detailed overview of the estimated conditional effects of the model (effects of the parameter employed corresponding to all reference conditions) can be found in Fig. 2D (see also Table A2). The results and statistical effects with regard to the individual experimental factors are described in the following.

**Table 2:** Model comparison of all Bayesian (GLM) models of experiment 2

Model	Fixed effects	Random effects	Pareto *k* check	LOOIC	BF
2A	*SCORE* ~ *NUM*	Random intercept for excerpt and participant	< 0.7	9763.5	1.67e + 06^*^
2B	*SCORE* ~ *TARGET*	Same as 2A	< 0.7	9761.3	1.84e + 06^*^
2C	*SCORE* ~ ILD:PRESENCE^***^	Same as 2A	< 0.7	9739.6	1.21e + 05^*^
2D	*SCORE* ~ *ILD:PRESENCE*** + ***TARGET*	Same as 2A	< 0.7	9738.8	4.03e + 08^*^
2E	*SCORE* ~ *ILD:PRESENCE*** + ***TARGET*** + ***NUM*	Same as 2A	< 0.7	9739.2	5.03e + 11^*^
2F	*SCORE* ~ *ILD:PRESENCE*** + ***NUM:PRESENCE*** + ***TARGET:PRESENCE*	Same as 2A	< 0.7	9734.5	1.54e + 17^*^
2G^**^	*SCORE* ~ *ILD:PRESENCE*** + ***NUM:PRESENCE*** + ***TARGET:PRESENCE*	Random slope and random intercept for excerpt and participant	< 0.7	9619	(Excluded)^**^

Model summary (fixed effects) shows how the model was passed to the brms package in R [**SCORE = **binary-coded MSA score; **NUM** = number of instruments in the mix; **ILD = **inter-aural level difference condition; **TARGET = **choice of the target instrument; **PRESENCE** = target instrument is part of the mixture. ‘**:**’ indicates an interaction effect; **LOOIC** = leave-one-out cross-validation information criterion; **BF** = Bayes factor]. ^*^Here we tested against a null model, which includes only random effects but no fixed effects. ^**^Model 2G indicated that the effective sample size (ESS) is too low, indicating that the MCMC algorithm used to estimate the posterior distributions of the model parameters may not have fully converged. A low ESS value can lead to biased or imprecise estimates. ^***^Given the design of the task, the **ILD** parameter cannot be interpreted without an interaction effect with the presence of the target in the mix (**PRESENCE**)

#### Level ratio between target and mixture

Descriptive statistics and overall results are presented in Fig. 2A. The model estimates that as the target-to-mixture level ratio was decreased, the MSA task became increasingly difficult. The median difference in accuracy when changing the level ratio from 0 dB (*M* = 93.6%; CI = [90%; 96.3%]) to − 5 dB (M = 89.8%; CI = [85.4%; 93.5%]) was 3.8 percentage points. Based on the B-GLMM, non-linear one-sided hypothesis testing was performed, indicating that the posterior probability (PP) negative difference between 0 dB and − 5 dB was above 0.95. This can be interpreted as evidence for a difference between conditions (for uniform priors, the posterior probabilities will exactly correspond to frequentist one-sided *p*-values; see, e.g., Marsman & Wagenmakers, 2017). A substantial difference in performance was also observed as the level ratio between the target and mixture was further decreased, with the detection rate dropping from a median percentage correct of 80.1% (CI = [73.3%, 85.5%]) at − 10 dB to 56.6% (CI = [50.5%, 64.2%]) at − 15 dB. When there was no target in the mix, the median detection accuracy was 79% (CI = [70.2%, 86.6%]). In short, the model yielded strong evidence that this difference was meaningful. The congruence between the descriptives of the observed data and the model estimates highlights the strong model fit and underscores the robustness of the observed effect.

#### Choice of the target instrument

The model indicates strong interaction effects of both the number of instruments in the mix and the target category with the presence of the target in the mix (***PRESENCE***). Thus, differences in correct detection rates must be interpreted according to this interaction. In line with previous research (e.g., see Bürgel et al., 2021), lead vocals yielded outstanding accuracy. Even though several items were excluded from this condition due to their perfect detection rates (100% of participants answered correctly), lead vocals still demonstrated the highest detection rates both when the target was presented in the mix (*M* = 87.2%; CI = [82.5%; 91.2%]) and when the target was not presented in the mix (*M* = 94.1%; CI = [88.7%; 97.0%]). The bass, on the other hand, was the most difficult to detect and also showed the greatest variation: for items with the target in the mix the median was 60.8% (CI = [54.3%; 68.0%]), and it was 80.2% (CI = [70.3%; 88.5%]) when the target instrument was not included. Both guitar (*M* = 84.8%; CI = [80.0%; 89.1%]) and piano (*M* = 87.1%; CI = [82.4%; 91.3%]) remained in an easy difficulty range for items in which the target was present, but became moderately difficult when the target was not present (i.e., *M*_guitar_ = 70.8%; CI = [61.1%; 80.5%] and *M*_piano_ = 70.2%; CI = [60.7%; 80.3%]). Similar to the ***LEVEL*** factor, non-linear hypothesis testing was performed. Non-negligible differences were found between the bass and all other target instruments, both when the items included the target in the mixture and when the target was missing. When the target was not part of the mixture, the lead vocals also showed substantial differences in detection rates compared to all other instruments. For items in which the target played in the mixture, the differences in performance for lead vocals were apparent only in comparison to the bass. Guitar and piano had comparable detection rates.

#### Number of instruments in the mixture

The B-GLMM indicated a relevant interaction effect for the number of the instruments and the presence of the target in the mixture. Overall, when the target did not play in the mixture, more complex musical excerpts with six instruments in the mixture showed lower detection rates (*M* = 74.1%; CI = [65.4%; 82.7%]) compared to simpler mixtures with three instruments (*M* = 83.6%; CI = [75.0%; 90.4%]). When the target was present in the mixture, the median detection accuracy was slightly different for both groups: 77.4% (CI = [72.2%; 82.5%]) for the six-instrument mix and 82.5% (CI = [77.4%; 87.3%]) for the three-instrument mix. Based on the model, this difference was found to be meaningful.

#### Individual differences factors

We assessed the relationship between the model-derived MSA scores, which include participant random intercepts adjusted for guessing and inattention parameters, and the GMS perceptual and training scores using Pearson's product–moment correlation. We found a marginal correlation between participants' model-based MSA scores and the musical training subscale of the GMS (*r* = 0.103, *p* = 0.008), and a slightly larger correlation between MSA scores and the musical perception subscale (*r* = 0.188, p < 0.001). Even though the inter-individual variation was quite large within each hearing group of subjects, we observed a decrease in performance with the self-reported degree of hearing impairment. Averaged across all items, the mean instrument detection accuracy decreased from 79.8% (CI = [78.7%; 80.9%]) for participants with no HI, to 73.9% (CI = [69.8%; 77.9%]) for individuals with mild HI, 77.1% (CI = [72.8%; 81.4%]) for those with moderate HI, 74.5% (CI = [69.3%; 79.6%]) for participants with severe HI, and 69.0% (CI = [53.2%; 84.9%]) for individuals with profound hearing impairment. Participants’ observed responses are displayed in Fig. 2E, F, G.

### Discussion

As hypothesised, the model estimates suggested all three employed parameters had a robust influence on the participants’ accuracy. Experiment 1 showed that the accuracy in the MSA test depends on the choice of the target instrument, the number of instruments in the mixture, and the level ratio between the target and mixture. Lead vocals had the highest detection rates, while the bass was the most difficult to process. The detection rates were lower for more complex musical excerpts with six instruments in the mixture compared to simpler mixtures with three instruments. As indicated by the sequential model comparison (see BF in Table 1), the effect of the level ratio between the target and mixture had the most substantial effect on the difficulty of the task, with the task becoming increasingly difficult as the target-to-mixture level ratio decreased. When included in the model, both the number of instruments in the mixture and the choice of the target instruments added comparatively smaller but non-negligible improvements to the predictive performance of the model. The degree of hearing impairment also showed a relationship with the MSA test, with a decrease in performance as the degree of hearing impairment increased. There was a weak correlation between simple MSA sum scores and musical training and a slightly larger correlation between MSA scores and musical perception. Even among the most highly musically trained participants in this sample, no ceiling effects could be observed, whereas some of the more severely hearing-impaired individuals performed at chance level (see Fig. 2E and G). This suggests that the test was challenging enough to provide meaningful results for individuals with prior musical expertise and is also likely sufficient to measure ASA performance in the context of complex multi-source music for individuals with both normal hearing and severe hearing impairments.

Experiment 1 showed the strongest effects for the factor of the level ratio between target and mixture. In experiment 2, we explored whether another important factor of music production, spatialisation in terms of a stereo image, proved to be similarly powerful for adjusting item difficulty.

## Experiment 2: Calibration phase—Part 2

### Methods

#### Stimuli

**Fig. 3:**
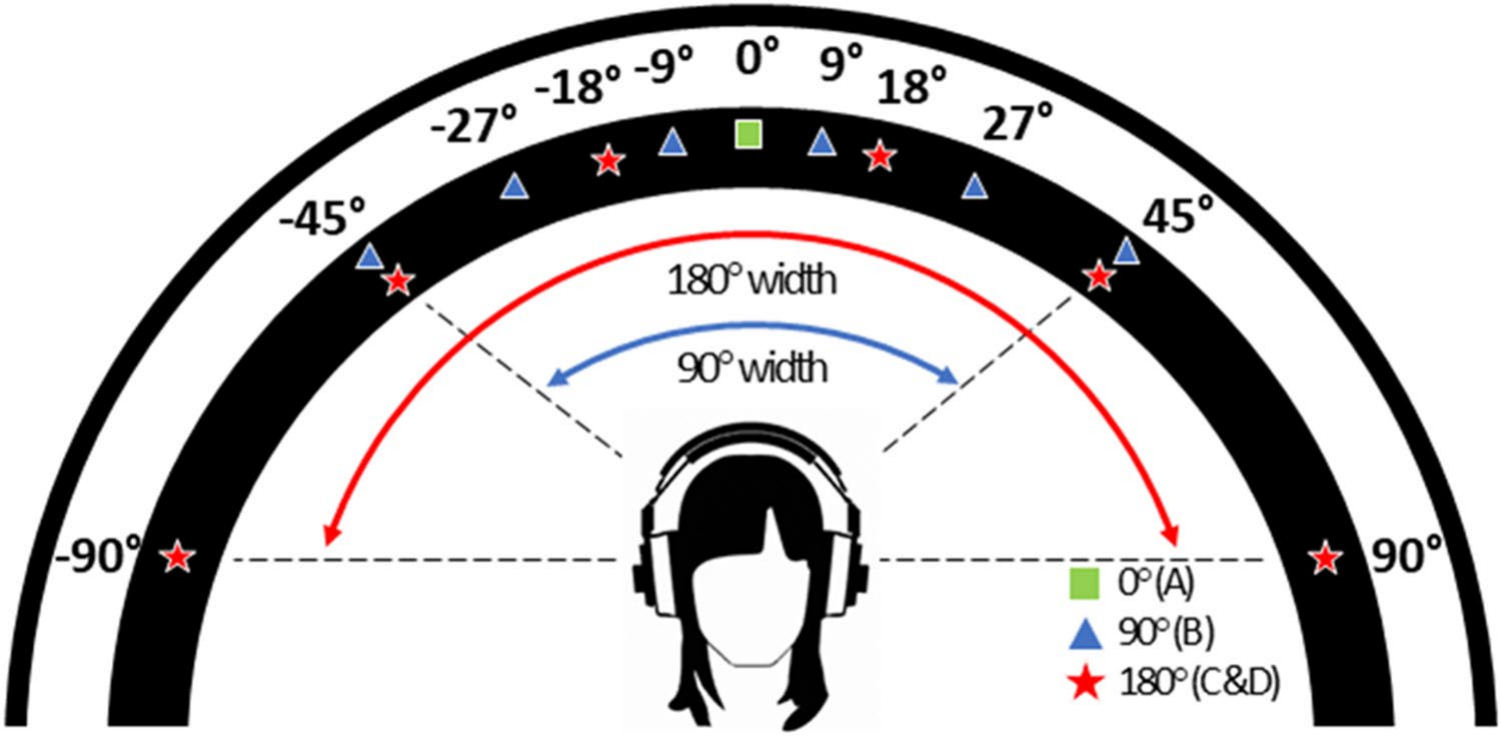
Schematic illustration of the stereo width conditions in frontal azimuth in experiment 2. (**A**) All instruments are presented at 0°, i.e., the monaural reference; (**B**) the instruments are distributed evenly at a stereo width of 90° (that is, each instrument is randomly allocated to one of the positions at − 45°, − 27°, − 9°, 9°, 27°, 45°); (**C**) all instruments were distributed evenly across the full stereo width of 180°; (**D**) all instruments were distributed evenly across the full stereo width of 180°, but the position of the target instrument was changed when it was presented during the target presentation phase compared to when it was presented within the mixture (during the mixture presentation phase)

In the second experiment, the same stimuli as in experiment 1 were used. For this experiment, however, instruments were presented spatially separated along the azimuth. To create the stereo image, inter-aural level differences (ILD) were adjusted. ILDs are critical binaural cues that contribute to sound source localisation (e.g., Stecker & Gallun, 2012). Here, we used the equal-power-panning method (e.g., Blauert & Braasch, 2008). Four conditions were thus generated, each characterised by varying panning widths, leading to stereo images with angular widths of (A) 0°, (B) 90°, and (C and D) 180°, yielding a total of 640 items (see Fig. 3 for a schematic illustration). Specifically, condition A served as a reference, where all instruments and the target were centrally localised. In condition B, both the target and the other instruments were randomly allocated to one of the positions at − 45°, − 27°, − 9°, 9°, 27°, or 45° (i.e., 90° of the frontal azimuth) within the stereo field. In conditions C and D, the positions were expanded to − 90°, − 45°, − 18°, 18°, 45°, or 90° (i.e., 180° of the frontal azimuth). In contrast to the fixed spatial positions of the target instrument in the preceding conditions, condition D introduced a modification: the spatial location of the target changed between its isolated presentation and its occurrence within the mixture. This alteration effectively removed the informative cue provided by a consistent target location. To avoid ceiling effects, the target-to-mixture level ratio was kept constant at − 10 dB in all conditions.

### Procedure

The procedure and materials used in this experiment were similar to those of experiment 1 and progressed in the following order: sound level calibration, Huggins headphone screening task, training phase of the MSA, data collection phase of the MSA, demographics questionnaire, and the two subscales for musical training and perceptual abilities of the German version of the Gold-MSI self-report questionnaire. Participants listened to all 160 audio excerpts, and for each excerpt, they were presented with one of the four different stereo width conditions. The order in which the excerpts were presented and the selection of the stereo width conditions were randomised across trials.

#### Participants

**Fig. 4:**
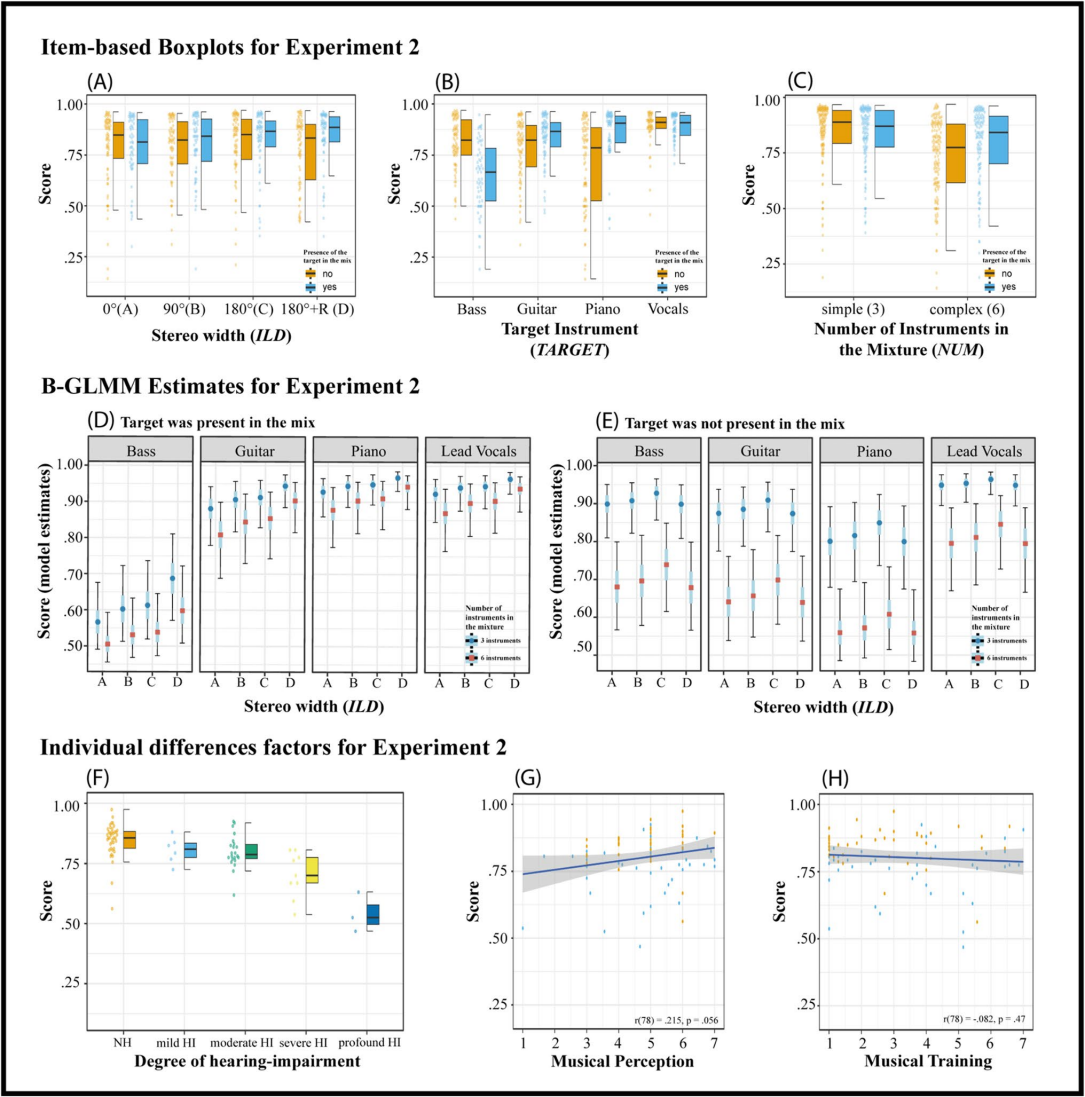
Results of experiment 2. Panels (**A**), (**B**), and (**C**) show the proportion of correct scores of individual test items for each of the parameters employed. Individual dots (left of the boxplots) represent the observed accuracy averaged over participants for each item. Light-blue boxplots correspond to items in which the target was present in the mixture. Panels (**D**) and (**E**) show the conditional effects plot for all conditions of the final B-GLMM (see Table 2). Median estimates with inner 50% (the length of the blue bars around the median) and outer 95% (the length of the black bars around the median) high-density intervals (HDI) of the estimated parameters are shown. HDIs are uncertainty intervals and reflect the probability of the parameter estimate being within the given intervals. Panel (**E**) shows the boxplots of participants’ average accuracy as a function of the degree of hearing impairment. Panels (**F**, **G**) show scatterplots illustrating the relationship between the participants' observed average detection scores and the two subscales of the Gold-MSI (musical training and musical perception). The individual dots represent the score for each participant averaged over all items

The second experiment included a total of 81 participants (53% female), of whom 40 were self-reported NH listeners and 41 self-reported HI listeners. One HI listener was removed from the analysis for stating that he did not use headphones. The remaining 80 participants were on average 43.2 years old (*SD* = 21.45) whereas NH individuals were predominantly younger, with a mean age of 26.4 years (*SD* = 8.4, range: 20–62 years), and the HI predominantly older, with a mean age of 60.2 years (*SD* = 17, range: 17–84 years). For a detailed overview with respect to the degree of hearing impairment, see Fig. 4F.

### Data analysis

#### Bayesian GLMM fitting

Similar to the first experiment, we used a Bayesian GLMM (Bernoulli family with an identity link; estimated using MCMC sampling with 12,800 observations, four chains of 6000 iterations, and a warmup of 3000) to predict participant performance. The strategy for building the random- and fixed-effects model structure was highly similar to experiment 1. However, instead of using the ***LEVEL*** parameter, the stereo width condition ***ILD*** (i.e., inter-aural level differences) with an interaction with the presence of the target in the mixture (***PRESENCE***), was included as fixed effect. First, we compared three models which each included one fixed effect only using Bayes factors (see Table 2). Model 2B, which included the ***TARGET*** as a fixed effect, provided the strongest evidence compared to the null model (BF = 1.84e + 06), followed by model 2A, which included ***NUM*** as a fixed effect (BF = 1.67e + 06), and model 2C, with ***ILD*** as fixed effects (BF = 1.21e + 05). Similar to experiment 1, these results emphasise the potential importance of the choice of the target instrument. In comparison, the effect size for ***ILD*** was the lowest.

For the model selection process, the same sequential comparison approach as in experiment 1 was used. Based on this approach, model 2F was selected as the best model, due to its balance between fitting performance and model complexity. Model 2G was not considered, as the effective sample size (ESS) was too low, indicating that the model did not fully converge. Model 2F included the factors ***ILD***, ***TARGET***, and ***NUM***, each with an interaction effect with ***PRESENCE***. Furthermore, a random intercept was included to account for the variation in the intercept across participants and excerpts, and the prior was defined as in experiment 1.

### Results

#### Model fit

The model (2F) converged (Ȓ < 1.01, ESS > 1000) and all Pareto *k* estimates were in an acceptable range (i.e., *k* < 0.5). Overall, the model was able to predict 79% of the trial-level observations. When excluding the random effects (random intercepts for excerpts and participants), the model could correctly identify 70.4% of the observed data. See Table A2 for a detailed model summary of the estimates and model fit. In line with the procedure in the first experiment, all items with 100% detection rates were excluded from the analysis (4 items with bass as target, 14 with guitar, 20 with piano, and 41 with lead vocals).

#### Stereo width

In this experiment, we investigated the impact of ***ILD*** on accuracy across four distinct stereo conditions. The B-GLMM identified a relevant interaction between stereo conditions and target presence within the mixture, emphasising the need to account for this interaction when interpreting the results. With the target absent from the mixture, median accuracy was as follows: 77.5% (CI = [67.8%; 86.0%]) for condition A (0°), 78.8% (CI = [68.9%; 87.1%]) for condition B (90°), 81.8% (CI = [72.3%; 89.4%]) for condition C (180°), and 77.4% (CI = [67.5%; 86.0%]) for condition D (180° + R). Accordingly, when the target was absent in the mix, a trend was present only from condition A to C (see Fig. 4E). Conversely, when the target was present in the mixture, a consistent trend in the median accuracy was apparent: Median accuracy was 79.5% (CI = [70.7%; 86.4%]) for condition A (0°), 82.1% (CI = [73.9%; 88.6%]) for condition B (90°), 82.8% (CI = [74.8%; 89.1%]) for condition C (180°), and 86.9% (CI = [79.9%; 92.2%]) for condition D (180° + R). In line with experiment 1, non-linear hypothesis tests were conducted, confirming a trend of improved accuracy. This improvement was most pronounced in the full stereo width conditions of 180° (C and D) in comparison to the monaural condition A (refer to Fig. 4D).

#### Choice of the target instrument

In experiment 2, we observed a similar pattern of interaction effects between the target instrument category and the presence of the target in the mix as in experiment 1. Consistent with our previous findings, lead vocals demonstrated exceptional accuracy (again, despite the exclusion of several items in this condition due to perfect detection rates). When the target was present in the mix, lead vocals showed a median detection rate of 92.3% (CI = [84.9%; 96.4%]), and when the target was absent, the rate was 88.3% (CI = [79.6%; 93.9%]). In contrast, the bass was the most challenging instrument to detect, with the largest degree of variability: the median was 58.1% (CI = [50.0%; 69.3%]) when the target was in the mix and 80.4% (CI = [70.3%; 88.5%]) when it was not. The guitar and piano demonstrated relatively low difficulty levels for items where the target was present, with median detection rates of 77.2% (CI = [67.1%; 86.2%]) for the guitar and 69.6% (CI = [59.5%; 79.8%]) for the piano. However, their difficulty increased moderately when the target was present (i.e., *M*_guitar_ = 88.2%, CI = [78.6%; 94.2%]; *M*_piano_ = 92.8%, CI = [85.7%; 96.6%]).

#### Number of instruments in the mixture

The results with respect to the number of instruments in the mix in experiment 2 were in line with those from experiment 1. More complex musical excerpts with six instruments in the mixture showed lower detection rates compared to simpler mixtures with only three instruments. When the target was not present in the mixture, the median for simpler mixtures with three instruments was 89.1% (CI = [80.4%; 94.5%]), while for more complex mixtures with six instruments, it was 68.6% (CI = [57.9%; 79.7%]). According to the model, this difference was considered considerable and meaningful. A similar trend, albeit less pronounced, was observed when the target was present in the mixture: The median correct detection rate was 85.5% (CI = [78.1%; 91.1%]) for the three-instrument mix and 80.2% (CI = [71.5%; 87.1%]) for the six-instrument mix.

#### Individual differences factors

The model indicated a decrease in accuracy with an increase in the degree of hearing impairment. On average, individuals with NH had a mean accuracy of 84.7% (CI = [82.5%; 86.9%]). The mean accuracy for individuals with mild HI was 80.5% (CI = [76.1%; 84.9%]), while for those with moderate HI it was 80% (CI = [77.1%; 82.9%]). For individuals with severe HI, the mean accuracy was 70.2% (CI = [64%; 76.4%]). The lowest mean accuracy was seen in individuals with profound HI, at 54.2% (CI = [44.8%; 63.5%]). The correlation between model-based MSA scores and the GMS musical training scores remained small (*r* =  − 0.09, *p* = 0.45), while the correlation with musical perception abilities was notably stronger (*r* = 0.20, *p* = 0.073).

### Discussion

Experiment 1 demonstrated that accuracy was dependent on the target instrument, with lead vocals yielding the highest and bass the lowest overall performance. Furthermore, mixture complexity influenced detection rates, as simpler three-instrument mixtures resulted in higher detection rates compared to more complex six-instrument mixtures. The results also suggested that individuals who reported better musical perceptual abilities tended to perform better on the MSA test, while the role of musical training remained inconclusive. Additionally, listeners with more severe hearing impairments exhibited lower MSA scores. Experiment 2 corroborated these findings.

We hypothesised that increased stereo width, as induced by ILDs, would facilitate target sound localisation and segregation within the mixture, leading to improved accuracy irrespective of the presence of the target. However, this was not the case when the target was absent from the mixture. One possible explanation is that ***ILD*** cues alone are not sufficiently strong to improve accuracy based on the stereo percept. This is unlikely, though, since we observed improved accuracy when the target was present in the mixture, particularly in stereo conditions C (180°) and D (180° + R) compared to monaural condition A. Interestingly, condition D displayed even higher accuracy, despite the target position changing between the presentation of the target alone and the mixture. This outcome was unexpected, and we initially assumed that changing the target position would cause confusion, leading to a decrease in detection rate in comparison to condition C. There are two possible explanations for this behaviour. First, participants were not informed about the cue, and the selection of conditions was fully randomised. Accordingly, participants might not have learned to rely on the positional cue due to the fully randomised and disclosed study design and thus never became confused by the change in position within condition D. However, this explanation cannot account for the improvement effect observed between conditions C and D. A second explanation might be the unexpectedness of the positional change of the target, which might have led to an acoustical novelty effect which shifted the cognitive locus of attention towards the unexpected stimuli (i.e., the target instrument at the unexpected position). Research has repeatedly shown that infrequent auditory changes in a series of otherwise repeated sounds trigger an automatic response to the novel or deviant stimulus (e.g., Parmentier, 2014), which supports this assumption. Further research is necessary to elucidate the underlying mechanisms responsible for these performance differences. Overall, the results indicate that increasing stereo width leads to comparably small improvements in accuracy (see BF for model 2C), but only for trials where the target is present in the mix.

## Integrated discussion: Calibration phase

Experiments 1 and 2 were designed to find suitable parameters to manipulate the test items’ difficulty to precisely probe MSA abilities of both NH and HI listeners, irrespective of age, musical sophistication, and musical training. We hypothesised that (1) the target-to-mix level ratio, (2) the choice of target instrument, (3) the number of instruments in the mixture, and (4) sound localisation cues would have a substantial influence on auditory scene analysis abilities in the context of music. We demonstrated that all four parameters exert an influence on scene analysis performance. Notably, despite the diversity in participants' musical perceptual abilities, prior musical training, and degree of hearing impairment, test scores still showed a reasonable degree of variability within groups, and we observed no strong ceiling or floor effects. The results of the two calibration experiments established conclusive evidence that all four parameters are suitable for manipulating test item difficulty.

Although the results identified four important parameters influencing the MSA test to a certain degree, we opted to include only the first three parameters in our test implementation. Our findings indicated only a modest effect of stereo width, i.e., ILDs, and only for those items that contained the target. By excluding the ILD parameter, we aimed to enhance the adaptability of our approach for online experimental setups utilising mono audio and to circumvent potential complications for participants with asymmetric hearing impairments. Moreover, as the perception of stereo width alone could contribute to making test items less difficult, and the other parameters were even more efficient for generating less difficult items, we chose to employ a more parsimonious model comprising only the three primary factors.

Based on the findings from experiment 1 and experiment 2, we can infer that individual differences in abilities play a notable role in the performance of the MSA task. The predictive accuracy of the final model, including both fixed and random effects, was consistently higher than the model with fixed effects alone. In experiments 1 and 2, the inclusion of random effects led to an increase in predictive accuracy of seven percentage points. This suggests that accounting for individual differences is crucial for understanding and predicting MSA performance. These results have significant implications for the development of adaptive MSA testing based on item response theory (IRT). By incorporating individual differences into the model, adaptive testing procedures can more efficiently estimate the underlying ability of participants and tailor the test items to their specific needs. This enables more precise and efficient measurement of MSA performance, while also reducing the likelihood of floor and ceiling effects that may be present in a one-size-fits-all approach. Furthermore, the model's strong predictive performance indicates that the selected factors adequately represent the underlying cognitive processes in an ASA task.

It is also important to acknowledge a few limitations of experiments 1 and 2. The assessment of participants’ degree of hearing impairment relied on self-report, and thus lacked accuracy. Another limitation is the online setting for testing: conducting listening tests remotely poses inherent challenges, such as potential variations in headphone quality and uncontrollable ambient noise, which can affect the reliability and accuracy of the assessments. Notably, the headphone screening test was not administered to participants with hearing impairments, leaving the presentation conditions uncontrolled. For instance, both the types of playback devices used and compliance with instructions to remove hearing aids remain unknown, thereby further complicating the evaluation of playback conditions. Overall, the challenges associated with conducting online listening tests highlight the importance of carefully considering the limitations and potential problems of these approaches when evaluating individuals with hearing impairments. In the validation experiment 3, we sought to address these limitations.

## Experiment 3: Test validation

The primary aim of experiment 3 was to validate an adaptive version of the MSA test under controlled laboratory conditions. The use of IRT models offers a flexible approach for creating an adaptive test. These models predict the probability of a correct response based on specific item parameters that capture different ways in which items might vary, such as item difficulty, item discrimination, and parameters for guessing and inattention. In this study, the B-GLMM used in experiment 1 can be considered an explanatory IRT model (Wilson & Boeck, 2004), as it describes the relationship between a person's latent trait level (i.e., the ability to identify musical instruments in a mixture) and their probability of responding correctly to test items. By incorporating item characteristics (e.g. target instrument, number of instruments in the mix, target-to-mixture level ratio, presence of the target in the mix) and person-specific random effects, the B-GLMM offers a flexible and robust approach to modelling response data. This makes it suitable for IRT applications in adaptive testing scenarios to assess the participants' ability to identify musical instruments within complex auditory scenes. Our IRT model then allows us to establish validity estimates of the adaptive MSA by examining its correlation with other measures of related constructs, such as speech-in-noise perception, melody discrimination, and mistuning perception. In addition to establishing validity, we sought to assess the test–retest reliability of the MSA test. One way to assess test–retest reliability within the IRT framework is to estimate the reliability both by using the standard error of measurements (SEM) derived from the model, and by comparing scores empirically from multiple adaptive MSA measurements within a single participant. We measured participants’ MSA ability twice on the same day to investigate test–retest reliability in controlled laboratory conditions, and also compared these measurements with a third set of MSA measurements, obtained through online testing on a different day in the participant's home environment.

To identify potentially important factors that might explain individual differences between test-takers, we also assessed working memory, musical sophistication, and individuals’ hearing thresholds as indicators of the degree of hearing impairment. This approach enables us to examine the test–retest reliability of the MSA and to pinpoint potential factors contributing to variations in participants' abilities across different musical perceptual domains. Subsequent analyses will scrutinise correlations among these diverse tests to elucidate the latent factors influencing musical perception.

### Methods

#### Test battery

##### Adaptive Musical Scene Analysis Test (MSA)

Based on the established B-GLMM of the first calibration experiment (model F), an adaptive version of the MSA was developed. Parameter estimates for the final model are given in Table A1. The final IRT model constitutes a classical four-parameter logistic model in which the discrimination parameter and the guessing and inattention parameter are constrained to be equal across all items. The item difficulty was estimated using the B-GLMM (model F), which applied the random-intercept mixed-effects structure for participant and excerpt, and by considering the fixed effects for (1) the choice of the target instrument, (2) the number of instruments in the mixture, and (3) the level ratio between the target and the mixture—each included in the model with an interaction with (4) the presence of the target in the mixture. From these results, an estimate of the level of difficulty for each of the 40 combinations of parameters was derived (see Fig. 2D). The B-GLMM fixed effects were converted to the metric of the desired item response model. By also incorporating the random-effect structure of the excerpts, we are also able to obtain a unique item difficulty estimate for each item. To set up the MSA task, we utilised the *psychTestR* (v2.23.0), an R package that provides the underlying testing mechanisms to create individual test packages that can be further employed in web-browser-based behavioural experiments. The adaptive version of the test was constructed using the psychTestRCAT (v1.6.0) package, which uses weighted-likelihood ability estimations of participant ability that range from approximately − 3 to + 3. The discrimination parameter was set to the standard deviation of the participant intercept (that is, the estimated median of the person random-effect structure). The adaptive item selection procedure of the MSA is constructed according to Urry's rule (as cited in Magis & Gilles, 2012). There, moderately difficult items are presented first to estimate participants' ability level using IRT (de Ayala, 2009). The first items eligible for selection have a difficulty level close to the item bank's mean difficulty, falling within a range of one standard deviation. Subsequently, more difficult items are presented to participants with higher ability and easier items to participants with lower ability. After each response, the participant's ability estimates are recalculated, and the next item selected is one that is closely aligned with their updated ability level. The same item is never presented twice. In this experiment, the test–retest reliability was established from the results of the adaptive test with a length of 30 items. For this experiment the MSA version 2.4 was used.

##### Oldenburger Satztest (OLSA; Kollmeier et al., 2015; Wagener et al., 1999)

The OLSA is an adaptive speech-in-noise perception test. The participants' task is to verbally repeat five-word sentences, spoken by a male speaker, embedded in speech-shaped white noise. The sentences were randomly generated from a predefined word class structure (name-verb-numeral-adjective-object), with each word class drawn from a pool of 10 possible word alternatives—resulting in sentences that were unpredictable for the listeners. While the masker level remained constant, the speech level was adaptively adjusted to determine the individual 50% speech-reception thresholds (SRT). To increase reliability, the composite scores of two test lists of 20 sentences each were used. The OLSA is essential for our experiment as it provides insight into the participant's speech perception abilities in a challenging listening environment, reflecting their ASA skills in the speech domain—measured by a well-established adaptive test. A relevant relationship between OLSA and MSA performance would support the notion that the two tasks tap into similar auditory scene analysis abilities.

##### Frequency discrimination task (FDT)

In this test, participants are presented with a series of three tones, one of which differs in frequency, and asked to identify the odd one out. Feedback on the correct response is provided on every trial. Using an adaptive 2-down-1-up procedure, the test score is calculated from two test sets of 40 items each, using the geometric mean of the last six reversal points within each set. In the context of the MSA task, the ability to discriminate frequencies allows participants to identify and segregate auditory streams based on their frequency content and thus helps to perceive and distinguish individual instruments within a complex musical scene. It is expected, therefore, that there would be a relationship between the FDT and MSA scores.

##### Melodic discrimination test (MDT; Harrison et al., 2017)

For each question of this adaptive melodic working memory test, participants hear three versions of the same unfamiliar melody. Each successive version is transposed to a semitone higher in pitch, and in one of these versions, a note has been altered. The task is to detect the ‘odd one out', while ignoring the transposition in pitch. By utilising weighted-likelihood ability estimations, the MDT uses a similar adaptive item selection procedure as the MSA. A default number of 20 items was used in this experiment. The MDT is important, as it evaluates a participant's working memory and their ability to discriminate auditory information, which relates to stages 2 and 3 of the MSA cognitive model. A strong correlation between MDT and MSA performance would indicate that working memory plays a role in the MSA task.

##### Mistuning perception test (MPT; Larrouy-Maestri et al., 2019)

This adaptive test assesses the ability to perceive mistuning in pieces of music. The task is to decide whether a vocalist is in tune or out of tune with the background music. The main output from the MPT is an ability score, corresponding to the ability estimate for the participant similar to the procedure mentioned for the MDT and MSA. A default number of 30 items was used in this experiment. As the MPT assesses a participant's ability to identify pitch deviations, it can inform their ability to perceive and segregate auditory streams (similar to the FDT), highlighting participants' sensitivity to tuning discrepancies. A relevant relationship between MPT and MSA performance would suggest that sensitivity to mistuning—that is, the sensitivity to frequency differences in the context of music—contributes to the MSA task.

##### Timbre perception test (TPT, Lee & Müllensiefen, 2020)

This test examines timbre perception abilities. Participants use a slider to best reproduce presented stimuli that vary along three important dimensions of timbre: temporal envelope, spectral flux, and spectral centroid. For each dimension, a total of six items was presented. The aggregated final estimate represents the average score of all three blocks, ranging from 1 to 100 (higher scores indicate better performance). The TPT relates directly to the participants' ability to perceive and segregate auditory streams based on timbre, a key aspect of the MSA task. A strong correlation between TPT and MSA performance would provide evidence for the importance of timbre perception in the MSA task.

##### Computerised Adaptive Beat Alignment Test (CA-BAT; Harrison & Müllensiefen, 2018)

The CA-BAT is an adaptive test of beat perception ability. This 2-AFC test assesses the ability to recognise the beat in a piece of music. Participants are presented with excerpts where they hear a piece of music together with a click track. The task is to decide whether the click track is on or off the beat in the music. The main output from the CA-BAT is an ability score, computed from the underlying item response model and corresponding to the ability estimate for the participant (similarly to the MDT). The full 25-item test with psychometric parameters and adaptive procedure identical to those of the original study was used. By making sense of the temporal structure of musical pieces, beat perception can provide the auditory system with prior knowledge about upcoming musical events, thereby guiding stream segregation. In this sense, the ability to perceive the beat helps listeners to organise auditory streams, making it easier to understand and follow the musical structure. A substantial relationship between CA-BAT and MSA performance would support the role of beat perception in the MSA task.

##### Backwards digit span memory test (BDS; e.g.Talamini et al., 2017; Weiss et al., 2016)

In this classical working memory test, participants remember sequences of digits. A test person is visually presented with digits one after the other and is then asked to recall the digits in the reverse order (e.g., for the sequence 1 2 3 4, the correct answer is 4 3 2 1). In total, two sequences with four digits (i.e., 2 × 4), 2 × 5 digits, 4 × 6 digits, and 4 × 7 digits were used. The final score represents the proportion of correctly recalled sequences. A strong correlation between BDS and MSA performance would further emphasise the importance of working memory in the MSA task.

##### Goldsmiths Musical Sophistication Index (GMS; Müllensiefen et al., 2014)

Instead of employing only the musical training and perception scale as in the calibration phase, the complete index (in German language) was used during the validation experiment. The final composite score ranging from 1 to 7, with 7 being the highest possible score, was generated for each subscale. A robust association between MSA performance and GMS scores would highlight the critical role of listeners' musical sophistication in ASA, whereas a relationship to the subscales that assess musical training and musical perceptual abilities would establish evidence in favour of the predictive validity of the test.

##### Pure-tone average audiometry (PTA)

For assessing the individuals' degree of hearing impairment, pure-tone audiometric thresholds were measured with an Interacoustics AD528 portable audiometer. The audiometer was used to measure thresholds at 0.125, 0.25, 0.5, 1, 2, 4, and 8 kHz using the standard clinical ascending–descending procedure in steps of 5 dB. Considering that elevated hearing thresholds in frequencies above 4 kHz have been shown to be meaningful in domains important for complex ASA listening scenarios (e.g., amplitude modulation detection thresholds and sound localisation; see, e.g., Moore, 2020; Narne et al., 2023), the PTA score was calculated as an average over all measured frequencies. When the PTA of the better ear exceeded 20 dB HL, participants were classified as HI (Humes, 2019). A relevant relationship between PTA and MSA performance would imply that hearing sensitivity plays a role in musical scene analysis.

### Procedure

Ethical approval was provided by the ethics committee at the University of Oldenburg. All participants provided written informed consent. The test was administered in two separate parts. The first part was conducted in a controlled laboratory setting, where participants were seated in a soundproof booth. The calibrated equipment consisted of a computer, an RME Babyface soundcard, and Sennheiser HD650 headphones. The long-term sound level was set to 75 dB SPL (A), measured with a Norsonic Nor140 sound-level metre using music-shaped noise as the excitation signal.

The first part of the test battery proceeded in the following order:

- Block 1:
  - Pure-tone audiometry.
  - Speech-in-noise (OLSA).
  - Frequency discrimination task.
- Block 2:
  - Demographics questionnaire.
  - Huggins headphone screening.
  - Mandatory pause of at least five minutes.
  - Training phase of the MSA.
  - First set of the adaptive MSA with 30 items.
- Block 3:
  - Melodic discrimination test.
  - Mistuning perception task.
  - Beat perception task.
  - Second set of the adaptive MSA with 30 items.

Between each of the assessment blocks, participants were given brief breaks that included verbal interactions and instructions from the experimenter. In addition, participants were encouraged to take as many breaks as necessary between tests to ensure that they were able to complete the tasks to the best of their ability. The order of the tests was carefully designed to maximise the effectiveness of the testing, taking into account aspects like test difficulty, potential learning effects, and cognitive fatigue. Each task was administered according to established protocols.

The second part of the test battery was performed online by the same group of participants, at least 24 h after the first part. After adjusting their volume to a loud but comfortable level, participants completed the following tests:

- Full Gold-MSI self-report questionnaire.
- Third set of the adaptive MSA with 30 items.
- Backwards digit span test.
- Timbre perception test.

This online component of the test battery allowed us to assess participants' MSA abilities in an online setting. The laboratory part of the study took approximately 80–120 min, whereas the online part took between 25 and 40 min. Because of missing data, some of the following analysis was conducted based on a subgroup of participants, which will be specified accordingly.

#### Participants

The experiment included a total of 74 participants (32 male and 42 female). The participants were divided into four groups: 30 older adults with NH (*M* = 63.2; *SD* = 7.2), 19 older adults with HI (*M* = 70.5; *SD* = 7.2), 24 younger adults with NH (*M* = 25.4; *SD* = 4.2), and one young adult with HI (age = 26). A graphical illustration of individuals' pure-tone audiometric thresholds can be found in Figure A1. Participants were recruited via a call for participation in the local newspaper in Oldenburg, Germany, and were compensated for their time (€10 per hour).

### Results

#### Test–retest reliability

**Fig. 5:**
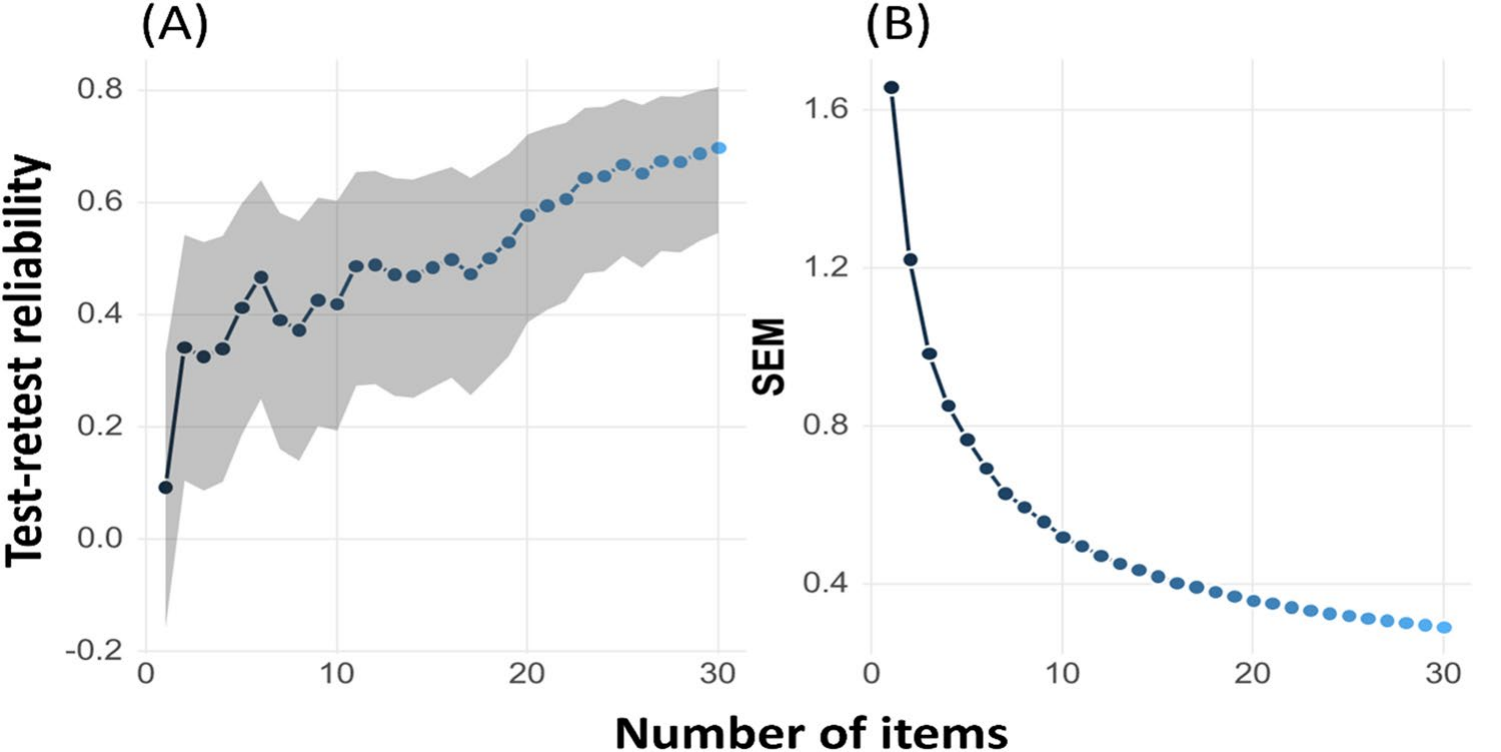
Test–retest reliability of the first and second set of the MSA as a function of test length is shown on the left and the standard error of measurement (SEM) as a function of test length on the right. The test–retest score (on the left) was assessed empirically and is based on the Pearson's product-moment correlation of all participants. The SEM (on the right) was derived from the model ability estimate

The two-part, two-day administration design of the study allowed us to assess the consistency of MSA performance over time and compare it in different playback environments. The test–retest reliability of the adaptive MSA was assessed using the intraclass correlation coefficient (ICC; see Koo & Li, 2016), using a two-way mixed-effects model with absolute agreement. For single measurements, which represent the reliability when the adaptive MSA was administered once, a moderate ICC(A,1) of 0.633 was observed (95% CI [0.46; 0.757], *F*(71,54.9) = 4.78, *p* < 0.001; Pearson’s *r*(70) = 0.67, *p* < 0.001). In contrast, the ICC for the mean of multiple measurements (that is, the average MSA score when the test was administered twice), improved to a substantial level with an ICC(A,2) of 0.775 (95% CI [0.625; 0.863], F(71,51.2) = 4.78, *p* < 0.001). Accordingly, the ICC indicates moderate to good consistency across the two sets of 30 items when administered under controlled conditions on the same day (see Fig. 5A). The results further suggest that the MSA shows moderately accurate and stable reliability values, even when comparing the combined MSA score of the controlled laboratory and the uncontrolled online environment (ICC(A,1) = 0.60, CI = [0.42; 0.73], *p* < 0.001; Pearson’s *r*(66) = 0.65, *p* < 0.001). As the length of the test increased, the model indicated a consistent decrease in the mean estimated standard error of measurement (SEM) for the MSA ability. This trend converged in a final SEM of 0.29 for the full test of 30 items, as illustrated in Fig. 5B. Averaged across all participants for whom complete data were available (*n* = 65), the median scores for the first, second, and third MSA item sets were 0.21 (95% CI = [0.17; 0.26]), 0.28 (95% CI = [0.22; 0.33]), and 0.33 (95% CI = [0.24; 0.38]), respectively. A repeated-measures ANOVA was conducted to assess the impact of training on test scores across three MSA test sets. The results suggest a small improvement in test scores due to training effects (*F*(2, 128) = 3.26, *p* = 0.042, η^2^ = 0.014).

#### Music-related and psychoacoustic tests

In the presented experiment, we found moderate correlations between the MSA test and timbre (*r* = 0.37), mistuning (*r* = 0.52), and beat perception (*r* = 0.46) scores (see Table 3). Notably, the strongest correlation was identified between the MSA and melody discrimination (*r* = 0.52). In addition, a moderate correlation was found between the MSA and speech-in-noise measures (i.e., SRT of the OLSA, *r*(63) =  − 0.39, *p* < 0.001) and after removing two severe outliers (that were minus three standard deviations from the median), the frequency discrimination task revealed a moderate association with the adaptive MSA (*r*(61) =  − 0.43, *p* < 0.001).

**Table 3:** The correlation of the adaptive MSA with selected tests from the test battery

	MSA online	SRT	FDT	MDT	CABAT	MPT	TPT	BDS	GMS general	GMS perceptual	GMS training	Age	PTA
*N*	66	72	72	72	72	72	66	66	66	66	66	72	72
Correlation	0.65**	− 0.45**	− 0.39**	0.52**	0.46**	0.52**	0.37*	0.36*	0.46**	0.37*	0.46**	− 0.4**	− 0.37*
95% CI	[0.49, 0.77]	[− 0.61, − 0.24]	[− 0.57, − 0.18]	[0.33,0.67]	[0.26,0.62]	[0.34,0.67]	[0.14,0.56]	[0.13,0.55]	[0.24,0.63]	[0.15,0.56]	[0.25,0.63]	[− 0.57, − 0.18]	[− 0.55, − 0.15]

Significance is denoted as *p < 0.01, **p < *0.001 (Pearson’s correlation). SRT corresponds to the speech-reception thresholds of the OLSA. For a complete correlation matrix see *Figure A2

#### Individual differences factors

Similar to the calibration experiments, we found a negative relationship between the degree of hearing impairment (measured by the individuals’ better ear elevated hearing thresholds) and MSA scores (*r* =  − 0.37). We also found a negative correlation between age and MSA scores (*r* =  − 0.40), however, both effects were confounded, as indicated by the correlation between age and hearing impairment (*r*(63) = 0.66, *p* < 0.001). Interestingly, MSA scores showed the highest correlations with age and pure-tone audiometric scores compared to all other employed music-related perceptual tests in the battery (see Figure A2 for a complete correlation matrix). This relationship gives a first indication of a potential use of the MSA as a relevant tool for diagnosing and profiling hearing impairment in a musical context. Nevertheless, the strongest relationship with PTA scores was observed with the OLSA speech-reception thresholds (*r* = 0.59; here, a positive correlation implies that a higher degree of hearing impairment leads to worse performance in the OLSA task). Participants' performance on the MSA tests demonstrated a notable correlation with BDS (*r* = 0.39), suggesting a moderate association with general working memory capabilities. Our findings further indicate a positive correlation between musical training and musical perception abilities with ASA abilities. Specifically, the correlation between the MSA test and musical training score was 0.46, and 0.37 for musical perception. The strongest correlations with the general musical sophistication score were found with mistuning perception scores (*r* = 0.65, *p* < 0.001), melody discrimination abilities (*r* = 0.53, *p* < 0.001), followed closely by the MSA (*r* = 0.48).

### Discussion

The aim of experiment 3 was to assess the psychometric properties of the adaptive version of the MSA test. Two reliability measures were calculated: the standard error of measurement (SEM) based on the ability estimate of the IRT model and the intraclass test–retest reliability. Both measures help to establish a comprehensive picture of reliability, as the SEM does not require participants to take the test twice, whereas the test–retest reliability does not rely on any model assumptions. Ideally, the optimal test length would be determined by the point at which both test–retest reliability and the SEM estimates plateau. However, in our study, this was not achieved with a test length of 30 items. The final test–retest reliability was acceptable and comparable to other previous measures of scene analysis ability (e.g., Kirchberger & Russo, 2015; Siedenburg et al., 2020). To further improve the test–retest reliability estimate, it is likely that increasing the test length would be necessary, although this remains to be seen in further empirical research.

To establish validity estimates, it is important to compare the MSA to other established tests that measure similar constructs. Unfortunately, there are no published tests specifically designed to assess ASA abilities with realistic musical excerpts. As a result, we must rely on comparisons with hypothetically associated psychoacoustic and music-related tests. The presented experiment found moderate correlations between the MSA test and various other music-related tests, such as timbre, mistuning, and beat perception, with the strongest correlation found with melody discrimination. The relationship between the MSA and these tests is likely due to shared underlying cognitive processes and sensory representations. For example, within the mistuning perception task, listeners need to have the ability to separate the vocal line from the accompaniment through auditory stream segregation, and then to assess the extent to which its pitch content conforms to the prototypical pitch distributions of the relevant musical style (Larrouy-Maestri et al., 2019). Just as in mistuning perception, the MSA task requires the separation of streams in the mixture. This similarity is also reflected in the comparable strengths of the relationship of the participants’ frequency discrimination scores to the MSA and those with MPT scores (see Figure A2). The moderate correlation between the MSA task and the frequency discrimination task also highlights that the MSA task is reliant on rather fundamental acoustical cues like pitch. Nonetheless, it also requires the ability to integrate multiple auditory cues and is affected by higher-level cognitive processes such as attention and memory. Accordingly, MSA and CABAT (beat perception) share the requirement to process and analyse temporal aspects of musical sound, specifically the rhythms and patterns within a piece.

Another important result is the substantial association between the MSA and the MDT, which assess the ability to discriminate between melodies. Melody discrimination abilities are often interpreted as reflecting more general cognitive traits and involve processes that are similar to those needed in the process of auditory scene analysis: in both tasks, musical streams have to be perceptually encoded, stored in memory (including melodic and timbral memory), then compared to each other, followed by a decision-making process. Timbre perception, on the other hand, is a multidimensional attribute which characterises the ability to discriminate between musical sounds, even when sounds are equal in loudness, tempo, and pitch. Accordingly, timbre plays a key role in the recognition of sound sources. Both the TPT and the MSA require the ability to identify and differentiate musical sounds based on their unique attributes.

In essence, the observed moderate relationships between the MSA test and specific music-related perceptual tasks—namely timbre identification, beat perception, melody discrimination, and mistuning perception—suggest that the MSA test measures similar but not the same cognitive and acoustic processing abilities. This provides compelling evidence for the task's convergent validity. Besides that, the moderate correlation between the MSA task and a speech-in-noise measure further supports its construct validity, as both concepts measure the ability to process and parse complex auditory information (speech vs music), separate relevant sounds from competing sounds (white noise vs a mixture of instruments), and identify and interpret different elements within an acoustic scene. To further establish the validity of the MSA task, future research could focus on demonstrating its predictive validity. For instance, researchers could compare the scores of highly trained musicians who are assumed to possess excellent auditory scene analysis skills (such as conductors) with those of musically untrained listeners. This would provide additional support for the MSA task as a valid measure of auditory scene analysis in music.

## General discussion

The objective of the current study was to establish a new test for ASA abilities in the context of music. Previous efforts to develop tests to assess musical perceptual abilities (Kirchberger & Russo, 2015; Siedenburg et al., 2020) were limited by relying on artificial stimuli and/or being unable to account for individuals with a broad range of perceptual abilities and diverse auditory profiles (e.g., hearing abilities, musical training, age, and cognitive abilities). In light of these limitations, our study aimed to develop an efficient and accessible test for ASA abilities in music. To accomplish this goal, we conducted three experiments.

In the first two experiments, we found that the target instrument, number of instruments in the mixture, target-to-mix level ratio, and presence of localisation cues (perception of stereo) had a relevant impact on an individual's ability to analyse and understand musical scenes. These results indicate that the specific characteristics of stimuli used in ASA tasks can greatly influence an individual's ability to analyse and understand musical scenes. To standardise the MSA test, we conducted a calibration (experiment 1 and experiment 2) with a sample of participants with varying degrees of hearing impairment and different musical backgrounds. In experiment 3, we built an IRT model based on the first three parameters of the already established B-GLMM. This allowed us to develop a reliable assessment tool for evaluating individuals' musical auditory scene analysis abilities. Our findings showed consistent MSA results when administering the test to the same individuals under different presentation conditions or to different individuals under the same conditions. We observed a moderate correlation between MSA and various aspects of musical perception, such as melody discrimination, timbre perception, mistuning perception, and beat perception. These findings emphasise the validity of the assessment and highlight the importance of considering ASA in understanding the interplay of the different facets of musical perception.

We also wanted to explore factors that might explain individual differences among test-takers, including musical background, working memory capacity, age, and degree of hearing impairment. Numerous studies have demonstrated that musical training or musical sophistication not only enhances basic perceptual abilities, such as pitch, timbre, and rhythm detection (Kannyo & DeLong, 2011), but also contributes to higher-order perceptual skills. These include musical imagery (Gelding et al., 2021), mistuning perception (Larrouy-Maestri et al., 2019), auditory tracking (Madsen et al., 2019), and auditory streaming segregation (Zendel & Alain, 2009). The results from experiment 3 align with this literature by showing a moderate relationship with musical scene analysis abilities. On the contrary, however, Bürgel et al. (2021) found no systematic difference between musicians and non-musicians using a similar instrument detection paradigm, which they attributed to the limited representation of musicians in their sample. Here, we recruited participants with a wide range of musical ability levels. We nonetheless observed only small correlations of musical training levels with MSA abilities in experiments 1 and 2, but moderate correlations in experiment 3, which remains intriguing. A potential explanation for these incompatible findings is the way in which the musical training score of the Gold-MSI is operationalised: the musical training score is more likely assessing a ‘lifetime’ training score, with the reported musical training potentially dating back many decades (e.g., ‘I have had formal training in music theory for __ years’). This could mean that someone who has intensively trained in music for the past 10 years may receive the same score as someone who played music extensively for 10 years, but has not played for the last 30 years—reducing the effect of prior musical training on musical perception performance due to decay. Recent musical training, in contrast, might have a stronger relationship with auditory scene analysis abilities than 'lifetime' musical training. Micheyl et al. (2006), for instance, showed that several hours of active training in a pitch discrimination task led non-musicians to achieve similar accuracy as musicians. This suggests that recent musical training might have a comparatively greater impact on ASA abilities. Accordingly, experiments 1 and 2 might have included ‘lifetime’ musicians, who had not played considerably in the recent past, while a higher proportion of actively performing musicians participated in the validation experiment. Certainly, this discrepancy must be addressed in future research.

Furthermore, our findings revealed a notable negative correlation between age and MSA scores and a negative association with the degree of hearing impairment. However, similar to Kirchberger and Russo’s (2015) study, the effect of the degree of hearing impairment was confounded by the factor of age. This makes it difficult to determine whether the observed correlation between the degree of hearing impairment and MSA performance is due to the hearing impairment itself or to factors associated with ageing. Goossens et al. (2017) used a matched-pair design to disentangle the effects of age and hearing impairment on speech perception. Participants were matched on age and then divided into groups based on the degree of hearing impairment, which allowed for a direct comparison of speech perception in individuals with similar age levels but different levels of hearing impairment. Goossens et al. (2017) also excluded participants with potential cognitive impairments. The results indicated that even when audiometric thresholds are within normal limits and individuals show no indication of even mild cognitive impairment, masked speech perception declines by middle age and further decreases with increasing age. However, since the prevalence of hearing-impaired participants was particularly low among the young age group, we were not able to achieve a fully matched-pair design. This is an area for future research. Overall, our findings converge with the notion that even fundamental aspects of music perception such as ASA appear to be affected by working memory, age, and the degree of hearing impairment (cf. Micheyl et al., 2013; Susini et al., 2023).

A notable limitation of the current study is its cultural specificity. The MSA test and its underlying theoretical frameworks are largely rooted in Western-centric music perception research. The chosen stimuli (base tracks and genre), target instruments, and the sample of participants mainly cater to Western audiences. This inherent cultural bias raises questions about the test's cross-cultural applicability and limits the generalisability of our findings. While it may be tempting to aim for a universally applicable MSA test, because of the complexity and diversity of musical experiences and traditions across cultures, it is more realistic and valid to develop culture-specific versions. For instance, a culture-specific version for Indian music could be developed by sourcing a multi-track database of Indian musical compositions and calibrating the test with Indian listeners. Although these culture-specific tests would feature different stimuli and item banks, they would remain conceptually comparable at the level of the latent variable—MSA ability. Such endeavours would make significant contributions to the field by broadening the scope of music perception research to be more inclusive and representative of global musical experiences (e.g., Jacoby et al., 2020).

Apart from cultural aspects, the test's ecological validity also merits acknowledgment. While the MSA test employs realistic musical stimuli, it should be noted that the listening conditions are nonetheless constrained by the experimental context. Specifically, the stimuli are presented over headphones in a controlled setting (and in a monaural configuration). This may not fully capture the complexities of auditory scene analysis as experienced in more naturalistic settings, such as live concerts, where various acoustic and attentional factors come into play. Therefore, the term 'ecologically valid' is used here with the understanding that the test conditions are a simplification of real-world musical experiences.

In summary, the newly developed adaptive MSA test can serve as a valuable contribution to music perception research by efficiently measuring ASA abilities in a realistic musical context for individuals with a broad range of dispositions. The MSA test is sensitive to the effects of the degree of hearing impairment (i.e., individuals with elevated hearing thresholds) and musical background (musical sophistication and training) but is not limited by it. Future extensions of the current item bank could incorporate excerpts of classical music or excerpts from other musical genres and cultures. The MSA test is an easy-to-implement, user-friendly, enjoyable, and efficient tool for evaluating ASA performance in the context of music. With a training and test time of less than eight minutes, it is quick to administer and allows for flexibility with adjustable test length (alongside known measurement accuracies) to meet the specific needs of the study or application. Additionally, it is open-source and freely available. Detailed information on the operational R package, including an online demo, can be found in our GitHub repository (https://github.com/rhake14/MSA). By studying ASA in the context of music, researchers can gain insights that can inform the development of new technologies and techniques for improving music perception in individuals with hearing impairment.

### Supplementary Information

Below is the link to the electronic supplementary material.

Supplementary file1 (DOCX 251 KB)

## Declarations

### Open practices statement

All program code of the final test (open-source version of the adaptive MSA) is available online at the reported URL (https://github.com/rhake14/MSA). All further data and program code will be made available on request.
