# Supplementary material for: Development of an adaptive test of musical scene analysis abilities for normal-hearing and hearing-impaired listeners
Source: Behav Res Methods. 2023 Nov 13;56(6):5456–81. doi: 10.3758/s13428-023-02279-y (PMC11335785; doi:10.3758/s13428-023-02279-y)
Supplement: Supplementary file 1 — Supplementary file1 (DOCX 251 KB) [file 13428_2023_2279_MOESM1_ESM.docx]

# Supplementary Materials

## Supplementary Figures


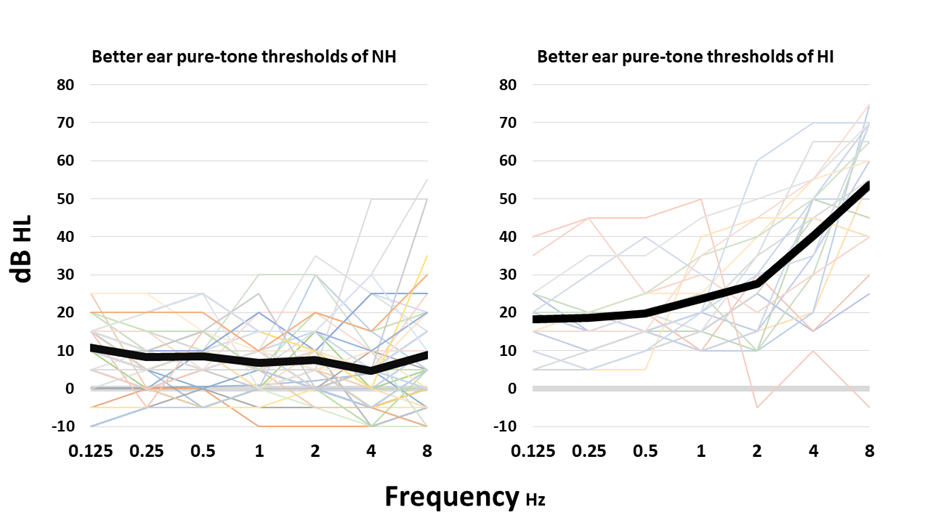
**Figure A1.** Graphical illustration of the pure-tone audiometric threshold (PTA) measurements of all participants for the respective better listening ear. Thresholds for normal-hearing individuals are plotted on the left and for hearing-impaired on the right. Standard clinical ascending-descending procedure in 5 dB HL steps were used. Participants were classified as hearing-impaired, when PTA over all frequency bins for the better ear exceeded 20 dB HL. The greyed lines show individual participants' threshold. The bold solid lines are representing the pure-tone average over all participants for each listening group.


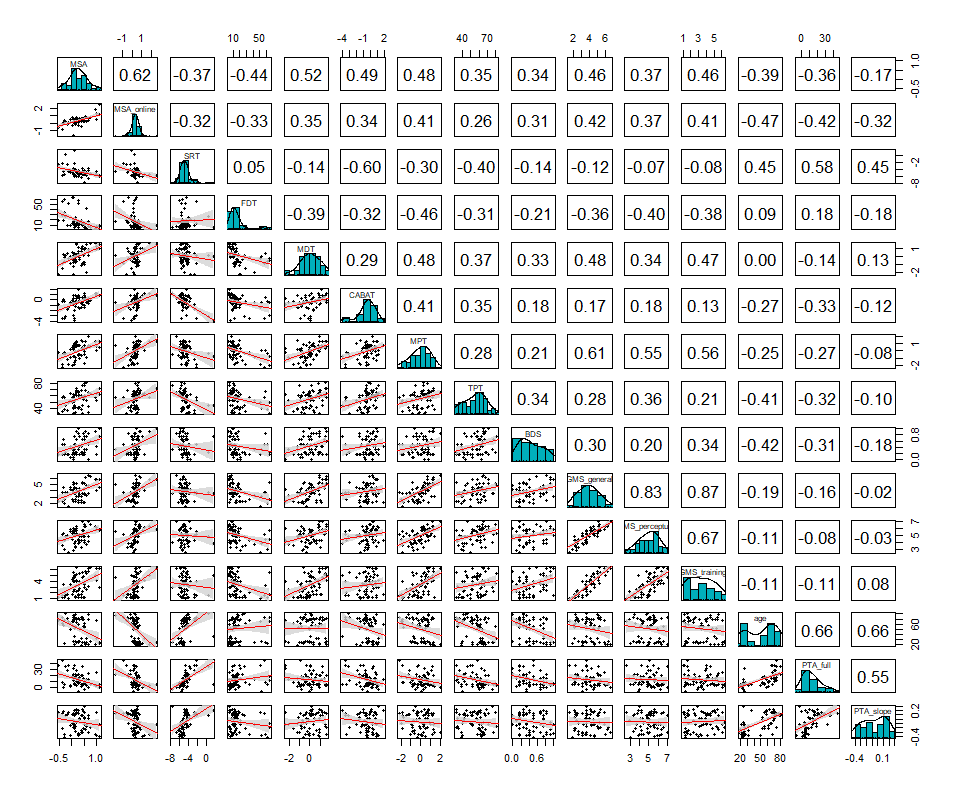
**Figure A2.** Correlation matrix of the adaptive MSA in relation to other psychoacoustic and musical-related tests. MSA is composite score of the first and second item set from the laboratory part of the experiment. MSA3 corresponds to a third test-set that was employed within the online testing environment. Subscales of the Gold-MSI (GMS), age and hearing impairment (PTA) are also included. Speech-reception thresholds (SRT) are the test results of the OLSA speech-in-noise test. For this matrix, extreme outliers and participants with missing data have been excluded (N = 63).

## Supplementary Tables

**Table A1.** Model summary of the final Bayesian Model (1F) of Experiment 1

| **Random Effects:** | | | Median | 95% HDI | pd | Rhat | ESS |  |
| --- | --- | --- | --- | --- | --- | --- | --- | --- |
| ~ excerpt (levels: 156) | | | 1.56 | [1.35, 1.79] | 1 | 1 | 1703 |  |
| ~ participants (levels: 657) | | | 2.08 | [1.88, 2.31] | 1 | 1 | 2003 |  |
|  |  |  |  |  |  |  |  |  |
| **Fixed Effects:** | | |  |  |  |  |  |  |
| ***LEVEL*** | ***PRESENCE YES*** | ***Level -15*** | -2.43 | [-3.20, -1.63] | 1 | 1.002 | 2549 |  |
|  |  | ***Level -10*** | -.00039 | [-.75, .77] | .5002 | 1.002 | 2511 |  |
|  |  | ***Level -5*** | 1.52 | [ .77, 2.29] | 1 | 1.003 | 2536 |  |
|  |  | ***Level 0*** | 2.33 | [1.58, 3.11] | 1 | 1.002 | 2561 |  |
|  | ***PRESENCE NO*** | ***Level -inf (no target in mix)*** | .21 | [-.78, 1.15] | .6642 | 1.001 | 2865 |  |
| ***TARGET*** | ***PRESENCE YES*** | ***BASS*** | -2.17 | [-3.14, -1.21] | .9998 | 1.002 | 2420 |  |
|  |  | ***GUITAR*** | .91 | [-.02, 1.82] | .9726 | 1.001 | 1870 |  |
|  |  | ***PIANO*** | 1.32 | [ .37, 2.23] | .9971 | 1.002 | 2119 |  |
|  |  | ***LEAD-VOCALS*** | 1.32 | [ .39, 2.23] | .9962 | 1 | 2219 |  |
|  | ***PRESENCE NO*** | ***BASS*** | .02 | [-1.01, 1.10] | .5192 | 1 | 2598 |  |
|  |  | ***GUITAR*** | -.7 | [-1.76, .37] | .9001 | 1.001 | 2408 |  |
|  |  | ***PIANO*** | -.72 | [-1.76, .35] | .9066 | 1.001 | 2465 |  |
|  |  | ***LEAD-VOCALS*** | 1.62 | [ .58, 2.69] | .9991 | 1 | 2834 |  |
| ***NUM*** | ***PRESENCE YES*** | ***3 instruments in the mix*** | .85 | [ .20, 1.53] | .9952 | 1.004 | 1397 |  |
|  | ***PRESENCE NO*** | ***3 instruments in the mix*** | .79 | [ .24, 1.35] | .997 | 1.005 | 2006 |  |
|  |  | ***guessing intercept*** | .43 | [ .41, .45] | 1 | 1.001 | 4238 |  |
|  |  | ***inattention intercept*** | .00249 | [ .00, .01] | 1 | 1 | 5570 |  |
|  |  |  |  |  |  |  |  |  |
| **Model fit:** | | | Estimate |  |  |  |  |  |
| **ELPD** | |  | -13645.2 |  |  |  |  |  |
| **LOOIC** | |  | 27290.45 |  |  |  |  |  |
| **WAIC** | |  | 27282.69 |  |  |  |  |  |
| **Sigma** | |  | 1 |  |  |  |  |  |
| **Log_loss** | | | 0.36 |  |  |  |  |  |

***Note.*** *Parameter column (most left) shows the interaction of the presence of the target (PRESENCE YES = target instrument is part of the mixture) and the three employed parameter: level-ratio between target and the mixture (LEVEL), choice of the target instrument (TARGET), and the number of instruments in the mix (NUM 3 = three instruments in the mix). The estimates are presented in log-scale format.*

**Table A2.** Model summary of the final Bayesian Model (2F) of Experiment 2

| **Random Effects:** | | | Median | 95% HDI | pd | Rhat | ESS |  |
| --- | --- | --- | --- | --- | --- | --- | --- | --- |
| ~ excerpt (levels: 160) | | | 1.63 | [1.40, 1.89] | 1 | 1 | 3267 |  |
| ~ participants (levels: 80) | | | 1.56 | [1.27, 1.90] | 1 | 1 | 2272 |  |
|  |  |  |  |  |  |  |  |  |
| **Fixed Effects:** | | |  |  |  |  |  |  |
| ***ILD*** | ***PRESENCE YES*** | ***ILD B (90°)*** | .11 | [ .17, .44] | .76 | 1 | 9398 |  |
|  |  | ***ILD C (180°)*** | .41 | [ .08, .72] | .99 | 1 | 8991 |  |
|  |  | ***ILD D (180° + R)*** | -.008 | [-.33, .30] | .52 | 1 | 8502 |  |
|  | ***PRESENCE NO*** | ***ILD B (90°)*** | .33 | [ .00, .62] | .97 | 1.001 | 8614 |  |
|  |  | ***ILD C (180°)*** | .38 | [ .05, .70] | .98 | 1 | 9091 |  |
|  |  | ***ILD D (180° + R)*** | .91 | [ .51, 1.25] | 1 | 1 | 8561 |  |
| ***TARGET*** | ***PRESENCE YES*** | ***BASS*** | .71 | [-.07, 1.45] | .96 | 1.003 | 2149 |  |
|  |  | ***GUITAR*** | .43 | [-.03, 1.19] | .86 | 1.001 | 1724 |  |
|  |  | ***PIANO*** | -.22 | [-1.01, .57] | .71 | 1.004 | 1987 |  |
|  |  | ***LEAD-VOCALS*** | 1.51 | [ .72, 2.30] | 1 | 1 | 2582 |  |
|  | ***PRESENCE NO*** | ***BASS*** | -1.37 | [-2.16, -.58] | 1 | 1 | 2010 |  |
|  |  | ***GUITAR*** | 1.05 | [ .29, 1.84] | 1 | 1.001 | 2084 |  |
|  |  | ***PIANO*** | 1.67 | [ .89, 2.43] | 1 | 1 | 2438 |  |
|  |  | ***LEAD-VOCALS*** | 1.59 | [ .80, 2.39] | 1 | 1 | 2834 |  |
| ***NUM*** | ***PRESENCE YES*** | ***3 instruments in the mix*** | -1.26 | [-1.80, -.74] | 1 | 1.001 | 2233 |  |
|  | ***PRESENCE NO*** | ***3 instruments in the mix*** | -.46 | [-.96, .06] | .96 | 1.001 | 2484 |  |
|  |  | ***guessing intercept*** | .41 | [ .40, .44] | 1 | 1 | 10165 |  |
|  |  | ***inattention intercept*** | .00199 | [ .00, .007] | 1 | 1 | 8578 |  |
|  |  |  |  |  |  |  |  |  |
| **Model fit:** | | | Estimate |  |  |  |  |  |
| **ELPD** | |  | -4867 |  |  |  |  |  |
| **LOOIC** | |  | 9734 |  |  |  |  |  |
| **WAIC** | |  | 9733.1 |  |  |  |  |  |
| **Sigma** | |  | 1 |  |  |  |  |  |
| **Log_loss** | | | 0.363 |  |  |  |  |  |

***Note.*** *Fixed effects column (most left) shows the interaction of the presence of the target (PRESENCE YES = target instrument is part of the mixture) and the three employed parameter: choice of the target instrument (TARGET), the number of instruments in the mix (NUM 3 = three instruments in the mix), and the stereo width condition, elicited by inter-aural level differences (ILD): (A) All instruments are presented at 0°, i.e. the monaural reference; (B) the instruments are distributed evenly at a stereo width of 90°; (C) the instruments are distributed at full stereo width of 180°; (D) similar stereo panorama as in condition C, but the target is presented at a different position when the target instrument is presented in isolation compared to the presentation phase when the whole instrument mixture is playing. The estimates are presented in log-scale format.*

**Table A3.** Model summary of the simple Bayesian Model (2C) of Experiment 2

| **Random Effects:** | | Median | 95% HDI | pd | Rhat | ESS |  |
| --- | --- | --- | --- | --- | --- | --- | --- |
| ~excerpt (number of levels: 156) | | 2.12 | [1.84, 2.47] | 1 | 1 | 2273 |  |
| ~participant (number of levels: 567) | | 1.55 | [1.27, 1.90] | 1 | 1 | 1720 |  |
|  |  |  |  |  |  |  |  |
| **Fixed Effects:** | |  |  |  |  |  |  |
| ***PRESENCE YES*** | ***ILD A (0°)*** | .61 | [ .06, 1.16] | .98 | 1.002 | 1041 |  |
|  | ***ILD B (90°)*** | .89 | [ .33, 1.43] | 1 | 1.002 | 1075 |  |
|  | ***ILD C (180°)*** | .98 | [ .42, 1.51] | 1 | 1.002 | 1081 |  |
|  | ***ILD D (180° + R)*** | 1.51 | [ .96, 2.06] | 1 | 1.001 | 1117 |  |
| ***PRESENCE NO*** | ***ILD A (0°)*** | .53 | [-.02, 1.05] | .97 | 1.006 | 1010 |  |
|  | ***ILD B (90°)*** | .63 | [ .06, 1.16] | .98 | 1.005 | 1044 |  |
|  | ***ILD C (180°)*** | .91 | [ .36, 1.44] | 1 | 1.006 | 1055 |  |
|  | ***ILD D (180° + R)*** | .49 | [-.07, 1.02] | .96 | 1.004 | 1047 |  |
|  | ***guessing intercept*** | .41 | [ .40, .44] | 1 | 1 | 7772 |  |
|  | ***inattention intercept*** | .00212 | [ .00, .01] | 1 | 1 | 7131 |  |
|  |  |  |  |  |  |  |  |
| **Model fit:** | | Estimate |  |  |  |  |  |
| **ELPD** |  | -4869.90 |  |  |  |  |  |
| **LOOIC** |  | 9739.80 |  |  |  |  |  |
| **WAIC** |  | 9738.17 |  |  |  |  |  |
| **Sigma** |  | 1 |  |  |  |  |  |
| **Log_loss** |  | .36 |  |  |  |  |  |

***Note.*** *Fixed effects column (most left) shows the interaction of the presence of the target (PRESENCE YES = target is part of the mixture) and the stereo condition, elicited by inter-aural level differences (ILD): (A) All instruments are presented at 0°, i.e. the monaural reference; (B) the instruments are distributed evenly at a stereo width of 90°; (C) the instruments are distributed at full stereo width of 180°; (D) similar stereo panorama as in condition C, but the target is presented at a different position when the target instrument is presented in isolation compared to the presentation phase when the whole instrument mixture is playing. The estimates are presented in log-scale format. The results illustrate the main effect for the ILD conditions.*

**Table A4.** Self-assessment of individual’ hearing impairment

| **Question** | | | | | | |  |
| --- | --- | --- | --- | --- | --- | --- | --- |
| Do you feel that you have a hearing loss? | | | | | | |  |
| (While this may not map onto your experience exactly, which of these best describes your hearing without hearing aids.) | | | | | | |  |
|  |  |  |  |  |  |  |  |
| **Options** | | | | | | |  |
| □ No, I do not feel that I have a hearing loss. | | | | | | |  |
| □ Yes, I have the feeling of being mildly hearing impaired (hearing loss of 20-40 dB):  When I am talking to a person in a quiet room, I can usually understand a conversation. In noisy situations (e.g. in a pub) and in group conversations, I sometimes have problems understanding speech. | | | | | | |  |
| □ Yes, I have the feeling of being moderately hearing impaired (hearing loss of 41-60 dB):  I have difficulty understanding speech and often have to ask others to repeat what they have said, both in face-to-face conversations and on the phone. I find it difficult to follow conversations in noisy situations. I have to turn up the TV and radio to hear them clearly. | | | | | | |  |
| □ Yes, I have the feeling of being severely hearing impaired (hearing loss of 61-80 dB):  I can barely understand speech without hearing aids and use lip-reading/sign language to assist communication. | | | | | | |  |
| □ Yes, I have the feeling of being profoundly hearing impaired (hearing loss bordering on deafness; 81-95 dB):  Ordinary conversations are unintelligible. I cannot hear shouted words and have to rely on lip reading and/or sign language to communicate. | | | | | | |  |
| □ I don’t know | | | | | | |  |

*Note. Individuals choosing ‘I don’t know’ has been categorized as normal hearing. The version presented to German speaking participants is presented in Table A5.*

**Table A5.** Self-assessment of individual’ hearing impairment for German participants

| **Question** | | | | | | |  |
| --- | --- | --- | --- | --- | --- | --- | --- |
| Haben Sie das Gefühl, dass Sie einen Hörverlust haben? | | | | | | |  |
| (Auch wenn dies nicht genau auf Ihre Erfahrung zutrifft, sollten Sie sich überlegen, welche dieser Angaben Ihr Gehör ohne Hörgeräte am besten beschreibt.) | | | | | | |  |
|  |  |  |  |  |  |  |  |
| **Options** | | | | | | |  |
| □ Nein, ich habe nicht das Gefühl einen Hörverlust zu haben. | | | | | | |  |
| □ Ja, ich habe das Gefühl, leichtgradig schwerhörig zu sein (Hörverlust von 20–40 dB): Wenn ich mit einer Person in einem ruhigen Raum spreche, kann ich normalerweise ein Gespräch verstehen. In lauten Situationen (z. B. in einer Kneipe) und bei Gruppengesprächen habe ich manchmal Probleme, Sprache zu verstehen. | | | | | | |  |
| □ Ja, ich habe das Gefühl, mittelgradig schwerhörig zu sein (Hörverlust von 41–60 dB):  Ich habe Schwierigkeiten, Sprache zu verstehen, und muss häufig andere bitten, das Gesagte zu wiederholen, sowohl im persönlichen Gespräch als auch am Telefon. In lauten Situationen fällt es mir schwer, Gesprächen zu folgen. Ich muss den Fernseher und das Radio lauter stellen, um sie deutlich zu hören. | | | | | | |  |
| □ Ja, ich habe das Gefühl, hochgradig schwerhörig zu sein (Hörverlust von 61–80 dB):  Ich kann Sprache ohne Hörgeräte kaum verstehen und benutze Lippenlesen/Gebärdensprache zur Unterstützung der Kommunikation. | | | | | | |  |
| □ Ja, ich habe das Gefühl, an Taubheit grenzend schwerhörig zu sein (Hörverlust von 81–95 dB):  Gewöhnliche Gespräche sind unverständlich. Ich kann geschriene Wörter nicht hören und muss mich auf das Lippenlesen und/oder Gebärdensprache verlassen, um mich zu verständigen. | | | | | | |  |
| □ Ich weiß nicht. | | | | | | |  |

*Note. Individuals choosing ‘Ich weiß nicht.’ has been categorized as normal hearing*.
